# Dispersive currents explain patterns of population connectivity in an ecologically and economically important fish

**DOI:** 10.1111/eva.13567

**Published:** 2023-06-21

**Authors:** Claire E. Schraidt, Amanda S. Ackiss, Wesley A. Larson, Mark D. Rowe, Tomas O. Höök, Mark R. Christie

**Affiliations:** ^1^ Department of Forestry and Natural Resources Purdue University West Lafayette Indiana USA; ^2^ Wisconsin Cooperative Fishery Research Unit College of Natural Resources University of Wisconsin‐Stevens Point Stevens Point Wisconsin USA; ^3^ U.S. Geological Survey Great Lakes Science Center Ann Arbor Michigan USA; ^4^ National Oceanographic and Atmospheric Administration National Marine Fisheries Service Alaska Fisheries Science Center Juneau Alaska USA; ^5^ NOAA Great Lakes Environmental Research Laboratory Ann Arbor Michigan USA; ^6^ Illinois‐Indiana Sea Grant Purdue University West Lafayette Indiana USA; ^7^ Department of Biological Sciences Purdue University West Lafayette Indiana USA

**Keywords:** gene flow, larval dispersal, oceanographic currents, population connectivity, yellow perch

## Abstract

How to identify the drivers of population connectivity remains a fundamental question in ecology and evolution. Answering this question can be challenging in aquatic environments where dynamic lake and ocean currents coupled with high levels of dispersal and gene flow can decrease the utility of modern population genetic tools. To address this challenge, we used RAD‐Seq to genotype 959 yellow perch (*Perca flavescens*), a species with an ~40‐day pelagic larval duration (PLD), collected from 20 sites circumscribing Lake Michigan. We also developed a novel, integrative approach that couples detailed biophysical models with eco‐genetic agent‐based models to generate “predictive” values of genetic differentiation. By comparing predictive and empirical values of genetic differentiation, we estimated the relative contributions for known drivers of population connectivity (e.g., currents, behavior, PLD). For the main basin populations (i.e., the largest contiguous portion of the lake), we found that high gene flow led to low overall levels of genetic differentiation among populations (*F*_*ST*_ = 0.003). By far the best predictors of genetic differentiation were connectivity matrices that were derived from periods of time when there were strong and highly dispersive currents. Thus, these highly dispersive currents are driving the patterns of population connectivity in the main basin. We also found that populations from the northern and southern main basin are slightly divergent from one another, while those from Green Bay and the main basin are highly divergent (*F*_*ST*_ = 0.11). By integrating biophysical and eco‐genetic models with genome‐wide data, we illustrate that the drivers of population connectivity can be identified in high gene flow systems.

## 1 INTRODUCTION

Identifying the spatial and temporal boundaries of freshwater and marine populations is critical for effective fisheries management and conservation (Begg et al., 1999; Carvalho & Hauser, 1995; Hixon et al., 2002). However, the delineation of aquatic populations can be challenging because many species are difficult to observe directly in their aquatic environments (Hedgecock et al., 2007). Furthermore, many fish and invertebrate populations are often connected by dispersal that occurs during a relatively cryptic pelagic larval stage throughout which most larvae are minuscule (~1‐5 mm) and are nearly transparent, making them difficult to observe directly. This pelagic larval stage is ubiquitous; many freshwater fishes that inhabit large lakes and over 95% of all marine fishes have a pelagic larval stage as part of their life histories (Nelson et al., 2016). Being pelagic and with limited swimming ability, larvae can be transported on currents to locations that are hundreds of kilometers away from where they were spawned (Christie, Johnson, et al., 2010, Christie, Tissot, et al., 2010; Cowen et al., 2006; Williamson et al., 2016). On the contrary, behavioral adaptations, homing mechanisms, and a complex interplay of biophysical processes (including currents) can result in individuals returning to the same site from where they were spawned (Almany et al., 2007; Christie, Johnson, et al., 2010, Christie, Tissot, et al., 2010; D'Aloia et al., 2015). Thus, identifying the role that currents play in connecting aquatic populations remains central to the effective conservation and management of aquatic ecosystems (Burgess et al., 2014; Liggins et al., 2019).

Both theoretical and empirical studies have demonstrated the importance of currents in defining population connectivity in aquatic systems (Cowen et al., 2007; Cowen & Sponaugle, 2009; Pineda et al., 2007; Selkoe et al., 2010; Treml et al., 2008). A smaller, but still substantial, number of studies have identified relationships between oceanic currents (including biophysical models parameterized by current data) and genetic differentiation (Galindo et al., 2006; Krueck et al., 2020; Legrand et al., 2022; Selkoe et al., 2016; Timm et al., 2020; White et al., 2010; Xuereb et al., 2018; reviewed in Jahnke & Jonsson, 2022). However, most of these studies have relied on current patterns from single points in time or that were averaged or integrated across weeks, months, and years (but see Krueck et al., 2020). Identifying the attributes and characteristics of currents that play a large role in defining population connectivity is critical for: (1) determining which currents to use for parameterizing connectivity matrices in theoretical or demographic models of population connectivity, (2) understanding the ecological (e.g., dispersal) and evolutionary (e.g., gene flow) linkages among populations in space and time, and (3) understanding patterns of genetic differentiation in aquatic systems. With respect to this last point, if dynamic and variable currents play a large role in determining patterns of genetic differentiation in aquatic systems, then this result could also help explain the “chaotic genetic patchiness” often described in marine systems. Chaotic genetic patchiness is commonly defined as unexpected patterns of genetic differentiation that are observed over small spatial scales and are not stable in time (sensu Broquet et al., 2013; Johnson & Black, 1982). Diverse and multifaceted drivers of chaotic genetic patchiness have been proposed, including genetic drift, high variance in reproductive success, and kinship (Iacchei et al., 2013). However, one relatively simple explanation for these patterns is that currents that connect local populations are highly dynamic and that currents from limited time periods are responsible for most of the dispersal among populations in a given year.

There are several challenges with identifying which currents serve as the primary drivers of population connectivity. First, high‐resolution oceanographic data and coupled biophysical models are required from numerous time points. Thus, high‐quality oceanographic data are requisite. Second, genetic differentiation is often used as a proxy for population connectivity, yet contemporary evolutionary processes (e.g., genetic drift) and past evolutionary legacies (e.g., signals of bygone selection) can confound genetic estimates of population connectivity (Waples, 1998; Waples & Gaggiotti, 2006; Whitlock & Mccauley, 1999). Third, there is no standardized approach for correlating genetic estimates of population connectivity with oceanographic‐based estimates of population connectivity (but see Krueck et al., 2020; White et al., 2010). One potential solution to this last challenge is to create species and system‐specific eco‐genetic agent‐based models (Dunlop et al., 2009) that can use connectivity matrices derived from oceanographic biophysical models as input and return pairwise genetic distance matrices as output (e.g., Krueck et al., 2020). With this approach, empirically derived estimates of genetic differentiation obtained from genotyping individuals from multiple sites (e.g., *F*_ST_) can be compared with eco‐genetic model‐derived estimates of genetic differentiation derived from spatially and temporally relevant oceanographic data. Here, we use this integrative approach across a 500 km latitudinal gradient to examine patterns of population connectivity in an ecologically and economically important fish species found throughout the Laurentian Great Lakes (hereafter Great Lakes).

In many ways, the Great Lakes have abiotic and biotic conditions that mirror temperate marine environments. High variability in nearshore currents, water temperature, and juvenile recruitment (Pritt et al., 2014) are some of the characteristics shared between these systems. Additionally, many Great Lakes fishes and invertebrates have a pelagic larval duration on the order of 30 to 40 days, high fecundity, high larval and juvenile mortality rates, and the potential for large population sizes—all characteristics shared with many marine species (Brazo et al., 1975; Forney, 1971; Ludsin et al., 2014; Pritt et al., 2014). Thus, insights gained from studies in the Great Lakes may be applicable to marine systems and vice versa. In this study, we focused on Lake Michigan yellow perch (*Perca flavescens*), an ecologically and economically important fish with an approximately 40‐day pelagic larval duration (Dettmers et al., 2005; Whiteside et al., 1985). Lake Michigan spans a latitudinal gradient of nearly 500 km from north to south and has two large embayments, Green Bay and Grand Traverse Bay. While circulation patterns may vary substantially on an interannual basis, some general patterns are manifest. For example, during summer months, cyclonic gyres often form in the southern portion of the basin and along‐shore currents from south to north dominate along the nearshore eastern portion of the lake (Beletsky et al., 2007; Höök et al., 2006). In the middle portion of the lake, the current patterns are often complex, but sometimes produce anticyclonic gyres that could act as barriers between southern and northern locations. Because substantial numbers of yellow perch larvae have been found in the middle of the lake (Dettmers et al., 2005), it is possible that these gyres could connect locations on the eastern and western sides of the lake. In the northern portion of the lake, the currents typically flow at much slower speeds than in the southern portion of the lake and the complex bathymetry and embayments may support comparatively isolated aggregations of yellow perch.

Previous genetics work has revealed that Great Lakes yellow perch have a shared evolutionary history (i.e., the Great Lakes may have been only colonized once). In fact, most yellow perch populations throughout the Great Lakes are dominated by a single mtDNA haplotype (Sepulveda‐Villet et al., 2009). However, nuclear loci illustrate that there is a clear separation of yellow perch populations among each of the Great Lakes (Sepulveda‐Villet & Stepien, 2012). Furthermore, the population connectivity of yellow perch within Lake Erie, which has been more extensively studied, demonstrates fine‐scale population genetic structure (Fraker et al., 2015; Sepulveda‐Villet & Stepien, 2011) and there is no a priori reason to suspect that the patterns of population connectivity would be any less complex in Lake Michigan. One canonical study examined five sites in Lake Michigan and found substantial genetic differences between Green Bay and Lake Michigan and that sites in southern Lake Michigan were genetically similar to one another (Miller, 2003). Nevertheless, fully characterizing the genetic structure of Lake Michigan yellow perch, as well as identifying the drivers of population connectivity in this system, has important implications for the successful conservation and management of species in this region and beyond (e.g., stock identification, delineation of management units, and identifying predictors of recruitment).

In this study, we sampled yellow perch from 20 sites circumscribing Lake Michigan. For every individual, we obtained genotypes at 9302 single‐nucleotide polymorphisms (SNPs) distributed throughout the yellow perch genome (Feron et al., 2020) using restriction site‐associated DNA sequencing (RAD‐Seq). We also used a Lagrangian particle tracking biophysical model to obtain high‐quality current‐derived connectivity matrices reflecting connections among 40 regions from 36 release dates spanning 6 years. Using data from both our empirical RAD‐Seq dataset and our integrated biophysical, eco‐genetic model we asked three questions: (1) What is the population structure of yellow perch throughout the main basin of Lake Michigan and Green Bay? (2) How much does variation in the pelagic larval duration, vertical behavior of larvae, local population sizes, and number of generations of gene flow explain the empirically derived estimates of genetic differentiation? and (3) How does spatial and temporal variation in currents (e.g., the release date used in the biophysical mode l) explain the empirically derived estimates of genetic differentiation? We find that there is substantial population structure between Green Bay and main basin perch populations and that population connectivity within the main basin is best explained by highly dispersive currents.

## 2 MATERIALS AND METHODS

### 2.1 Study species and sample collection

Yellow perch remain one of the most ecologically and economically important species throughout the Great Lakes. They are an abundant nearshore fish species, serve as important predators of small fishes and invertebrates, and are themselves important prey for larger fishes (Evans, 1986). Historically, yellow perch supported commercially important fisheries throughout the Great Lakes region; in Lake Michigan alone, peak annual commercial harvest would now represent close to $16 million (US$) in dockside value and much more at retail. However, Lake Michigan yellow perch populations began declining during the late 1980s and early 1990s which led to closures of most yellow perch commercial fisheries. Relevant life history characteristics for yellow perch populations include: a pelagic larval duration on the order of 30 to 40 days (Dettmers et al., 2005; Whiteside et al., 1985), high fecundity (~ 10,000 to 150,000 eggs/female), and type III survivorship (i.e., high mortality during early life stages; Brazo et al., 1975; Forney, 1971). Yellow perch typically spawn during late spring to early summer when currents within Lake Michigan are often at their weakest (Beletsky et al., 1999). Adult yellow perch have modest home ranges and mark–recapture studies have demonstrated that most adult yellow perch and their congener, Eurasian perch (*Perca fluviatilis*), have high site fidelity, particularly with respect to spawning grounds (Bergek & Björklund, 2009; Böhling & Lehtonen, 1984; Glover et al., 2008; Schneeberger, 2000). Thus, the predominant form of population connectivity for yellow perch likely occurs during the pelagic larval stage. Similar to many marine fishes, yellow perch larvae may have some control over their dispersal trajectories simply by varying their vertical position within the water column (Graeb et al., 2004; Leis, 2006). As the larvae develop, they may also become better at swimming, such that a combination of active and passive dispersal mechanisms may ultimately dictate where individual yellow perch are located when they transition to a demersal life stage. Because yellow perch have many ecological and life history characteristics in common with marine fishes, other Great Lakes fishes, and other commercially important fishes (Ludsin et al., 2014; Pritt et al., 2014), they represent an excellent model system for studying patterns of population connectivity in aquatic systems.

**FIGURE 1:**
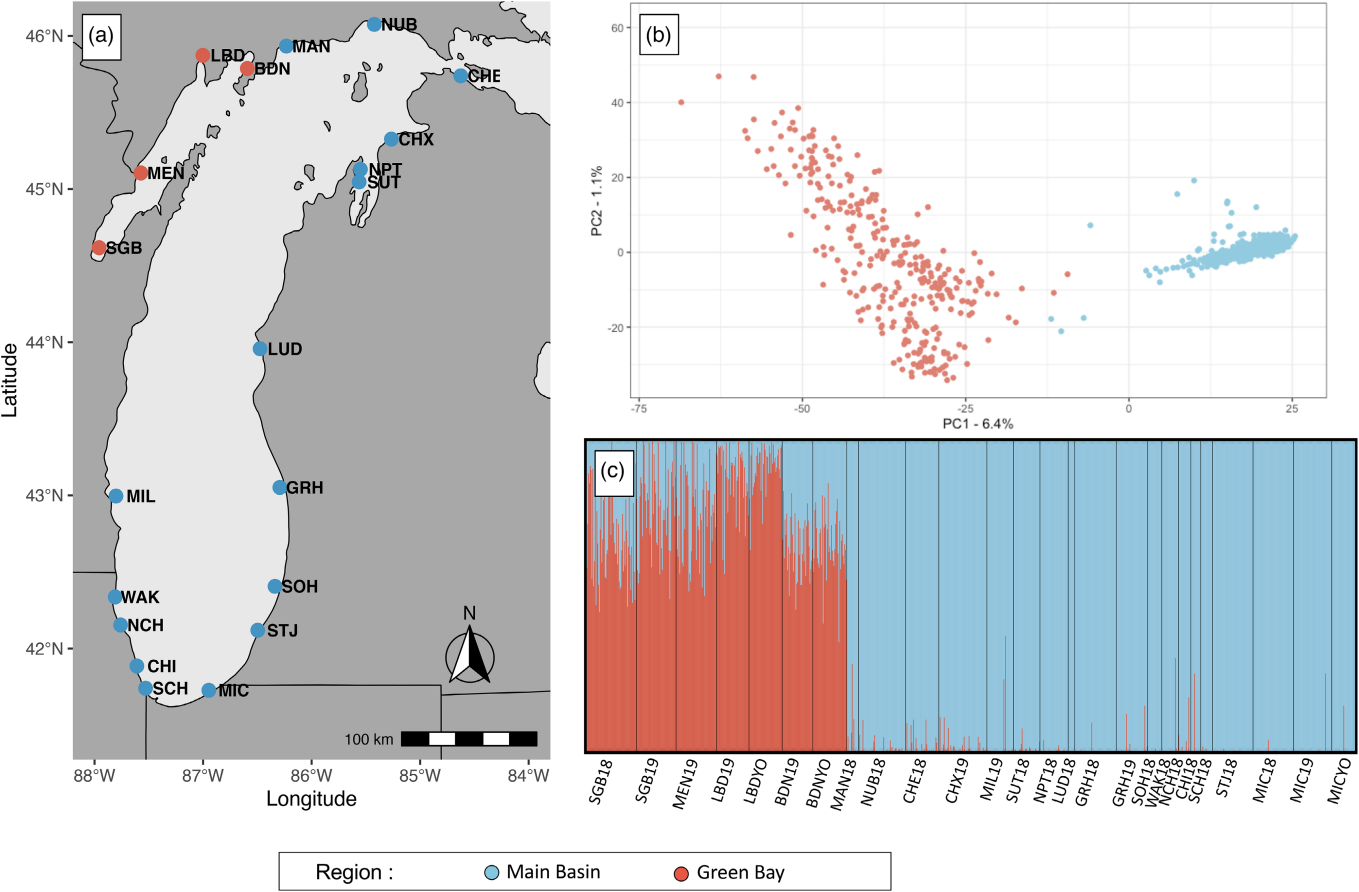
Sample collection sites and regional patterns of genetic differentiation. (a) A total of 959 yellow perch (*Perca flavescens*) were collected and genotyped from 20 sites, representing 26 collections (Table 1), circumscribing Lake Michigan. (b) Principal component analysis (PCA) for all genotyped individuals illustrates substantial genetic differences between Green Bay and main basin yellow perch. Also notice the larger spread of Green Bay individuals along axis 2, which suggests more variation among sites within Green Bay than sites within the main basin despite the much larger size of the main basin. (c) Results from STRUCTURE further illustrate the large genetic differences between Green Bay and main basin yellow perch where the proportion of Green Bay (red) or main basin (blue) ancestry is depicted for every individual as a single vertical line. Mean pairwise *F*_*ST*_ between Green Bay and main basin sites is equal to 0.11. The key to site name abbreviations is provided in Table 1.

Yellow perch were collected from 20 sites circumscribing Lake Michigan and Green Bay in 2018 and 2019 (Figure 1a; Table 1). In 2018, adults (i.e., individuals >100 mm in total length; see Table S1 for size data) were sampled using 12‐h overnight multimesh gill net sets. Sampling during the 2018 season began in southern Lake Michigan in early March and continued through the end of July at the northernmost sites to account for variation in regional spawning times. Young‐of‐year fish (i.e., juveniles) were sampled in September using beach seines. Individuals were classified as young‐of‐year based on size (<100 mm total length). Sampling in both 2018 and 2019 was supplemented with assistance from agency partnerships, specifically the Michigan Department of Natural Resources, the Indiana Department of Natural Resources, the Wisconsin Department of Natural Resources, the Illinois Natural History Survey, and the Grand Traverse Band of Ottawa and Chippewa Indians, where sampling methods consisted of bottom trawls, gill netting, and creel surveys. Small portions of fin tissue (~2 × 2 cm) were collected from every individual and stored in 95% nondenatured ethanol. A total of 1376 individuals were sampled over 2 years from which a subset of 959 samples that maximized representation among collection sites were selected for genotyping (Tables 1 and S1). Tissues were stored at −20°C upon arrival at Purdue University and held until DNA extraction.

**TABLE 1:** Sampling details for Lake Michigan yellow perch including collection site names, latitude, longitude, site name abbreviations (“Site ID”), region, year collected, age collected (yoy = “young of year”), and the number of individuals genotyped. A single individual from Algoma, Wisconsin was also genotyped but not used in any subsequent analyses (not shown). Locations are illustrated in Figure 1.

Site name	Latitude	Longitude	Site ID	Region	Year collected	Age collected	Number sequenced
Michigan City	41.72750	−86.94735	MICYO	Main Basin	2018	Yoy	28
			MIC19	Main Basin	2019	Adult	46
			MIC18	Main Basin	2018	Adult	50
Saint Joseph	42.11914	−86.49644	STJ18	Main Basin	2018	Adult	50
South Haven	42.40596	−86.33752	SOH18	Main Basin	2018	Adult	18
South Chicago	41.74067	−87.53000	SCH18	Main Basin	2018	Adult	16
Chicago	41.88567	−87.61000	CHI18	Main Basin	2018	Adult	13
North Chicago	42.15350	−87.76000	NCH18	Main Basin	2018	Adult	15
Waukegan	42.38700	−87.78000	WAK18	Main Basin	2018	Adult	20
Grand Haven	43.05141	−86.29291	GRH19	Main Basin	2019	Adult	38
			GRH18	Main Basin	2018	Adult	50
Ludington	43.95713	−86.47504	LUD18	Main Basin	2018	Adult	10
Milwaukee	42.99538	−87.80253	MIL19	Main Basin	2019	Adult	32
Suttons Bay	45.04650	−85.56199	SUT18	Main Basin	2018	Adult	32
Northport	45.12820	−85.55028	NPT18	Main Basin	2018	Adult	34
Charlevoix	45.32571	−85.26504	CHX19	Main Basin	2019	Adult	59
Cheboygan	45.73871	−84.62552	CHE18	Main Basin	2018	Adult	40
Naubinway	46.07465	−85.42233	NUB18	Main Basin	2018	Adult	72
Manistique	45.93287	−86.23245	MAN18	Main Basin	2018	Adult	15
Big Bay de Noc	45.78659	−86.59284	BDNYO	Green Bay	2019	Yoy	41
			BDN19	Green Bay	2019	Adult	40
Little Bay de Noc	45.87262	−87.00212	LBDYO	Green Bay	2019	Yoy	41
			LBD19	Green Bay	2019	Adult	40
Menominee	45.10461	−87.57281	MEN19	Green Bay	2019	Adult	49
South Green Bay	44.61706	−87.95805	SGB19	Green Bay	2019	Adult	50
			SGB18	Green Bay	2018	Adult	60
						Total =	959

### 2.2 Molecular methods

DNA was isolated from fin tissue with Qiagen DNeasy® Blood & Tissue Kits. Following an overnight (~14‐h) tissue digestion with Proteinase K incubated at 56°C, DNA was extracted in plates using standard kit protocols and eluted from the silica membrane with 200uL Tris Low‐EDTA buffer. Extractions were quantified using a Quant‐it™ PicoGreen® dsDNA Assay (Invitrogen), and DNA was normalized to a quantity of 200 ng or approximately 20 ng/μL.

Libraries for restriction site‐associated DNA (RAD) sequencing were prepared following the BestRAD protocol (Ali et al., 2016). Normalized DNA was digested with the restriction enzyme *SbfI* followed by ligation with indexed adaptors. Barcoded libraries were pooled into master libraries of 96 individuals and fragmented to ~300–500 bp with 12 30s cycles in a Q500 sonicator (Qsonica). Fragmented DNA was bound to Dynabeads™ M‐280 Streptavidin magnetic beads (Invitrogen) and washed with buffer to remove nontarget fragments. Following purification with AMPure XP beads (Beckman Coulter), master libraries were passed in series through the NEBNext® Ultra™ DNA Library Prep Kit for Illumina® at the End Prep step for (1) end repair and ligation of master library barcodes, (2) a 250‐bp insert size selection, and (3) a 12‐cycle PCR enrichment. Successful size selection and enrichment were confirmed with visualization of products on a 2% agarose E‐Gel (Invitrogen). Products underwent a final AMPure XP purification clean‐up followed by quantification with a Qubit® 2.0 Fluorometer. A total of 10 master libraries, each containing 96 individually barcoded samples, were sent to Novogene (Sacramento, CA) for PE150 sequencing on one lane of the Illumina Novaseq S4 platform.

Raw Illumina RAD sequence reads were processed using the STACKS v2.54 (Rochette et al., 2019) software pipeline. Reads were cleaned and demultiplexed by barcode using the STACKS subprogram process_radtags. Sequences were demultiplexed by barcode, filtered for Illumina quality score and enzyme cut‐site, and trimmed to 140 base pairs to reduce tail‐end sequencing errors (parameter flags = −‐filter_illumina, −‐bestrad, −t 140). The resulting filtered, individually assigned reads were aligned to the yellow perch reference genome (*P. flavescens* PFLA_1.0 assembly, GenBank accession GCA_004354835.1; Feron et al., 2020) with bowtie2 (Langmead et al., 2019; Langmead & Salzberg, 2012) (parameter flag = −‐very‐sensitive). Single‐nucleotide polymorphisms (SNPs) were called from reference‐aligned paired‐end reads with the STACKS subprogram gstacks (parameter flag = −‐rm‐unpaired reads), and individuals were genotyped at each identified SNP. The gstacks output files, which contain consensus sequences at each identified locus, as well as individual genotype data, were filtered through the STACKS subprogram “populations.” SNPs that were genotyped in less than 30% of individuals were discarded (parameter flag = −r 0.3), and results were exported in variant call format (VCF). SNPs were then filtered using vcftools v0.1.9 (Danecek et al., 2011). Post‐STACKS filtering largely followed the same workflow published in Gehri et al., 2021. Briefly, filtering consisted of (1) removing SNPs that were genotyped in fewer than 70% of individuals (Figure S1), (2) filtering out individuals with >70% missing loci, and (3) removing loci with a minor allele frequency (maf) of less than 0.01. We next used HDPlot (McKinney et al., 2017) to remove loci with a read ratio deviation greater than 5 and less than −5. A custom python script was then used to select SNPs with the highest allele frequency at each position. The resulting vcf file was converted to GENEPOP and STRUCTURE format using PGDSpider (Lischer & Excoffier, 2012).

### 2.3 Population genomics

Collection site summary statistics including observed heterozygosity (*H*_*O*_) and expected heterozygosity (*H*_*E*_) were calculated in R 4.0.2 (R Core Team, 2021) using the package adegenet v.2.1.1 (Jombart, 2008). Allelic richness (*Ar*) was calculated using the R package hierfstat v0.5.7 (Goudet, 2005). We used the R package HardyWeinberg (Graffelman, 2015) to test for deviations from Hardy–Weinberg Equilibrium using exact tests on each locus within each collection site. A Bonferroni correction based on the number of polymorphic loci within each collection site sample was used to identify loci out of equilibrium.

Cluster analyses were performed to identify population clusters and examine genetic similarity of individuals from all sites as well as separately within the main basin and Green Bay populations. The Bayesian clustering method implemented in STRUCTURE v2.3.4 (Pritchard et al., 2000) was first used to determine population structure present among all sampled locations (Green Bay and the main basin combined). For STRUCTURE analyses with all samples, the optimal inferred cluster (K) was determined using the delta K method (Evanno et al., 2005). Runs consisted of an initial burn‐in period of 50,000 Markov Chain Monte Carlo (MCMC) iterations followed by 50,000 iterations for each inferred cluster. Analyses were performed with K = 1–30 clusters and replicated five times for each value of K. Additional, runs investigating population structure were performed separately for both Green Bay and the main basin, respectively. For the Green Bay and main basin STRUCTURE analyses, we employed admixture and correlated allele frequency models, as this approach is most appropriate when subtle population structure is expected (Falush et al., 2003; Hubisz et al., 2009). Analyses were performed for K = 1–20 for the 19 main basin sites and for K = 1–8 for the 7 Green Bay sites, representing the total number of collection sites plus one, and replicated five times for each inferred cluster. As above, all runs consisted of an initial burn‐in period of 50,000 MCMC iterations followed by 50,000 iterations for each inferred cluster. Due to the appearance of admixed individuals between the two regions, NewHybrids (Anderson, 2003) was also run to further investigate whether any individuals might be F_1_ hybrids between Green Bay and the main basin. Five replicate runs of NewHybrids were performed with an initial burn‐in of 100,000 followed by 1,000,000 MCMC iterations. Posterior probabilities of group membership to parental group 1, 2, or F1 hybrids were averaged for each individual.

Pairwise *F*_*ST*_ between populations and 95% confidence intervals (calculated via bootstrapping, *n* = 1000) were calculated in hierfstat v0.5.7 (Goudet, 2005). Pairwise *F*_*ST*_ values were output as a genetic distance matrix and exported for analysis in GenAlEx v6.5 (Peakall & Smouse, 2006), where principal coordinate analysis (PCoA) was performed to visualize genetic differentiation among populations. Principal components analysis (PCA) was used to further support the results of both STRUCTURE and PCoA for both the Green Bay and main basin populations. Allele frequencies were scaled using the scalegen function in adegenet, and PCAs were run using these scaled matrices with the base R function prcomp. For the STRUCTURE and PCA analyses, we used the LD. thin function in the R package gaston 1.5.7 (Perdry & Dandine‐Roulland, 2020) with a threshold of 0.1 and max.dist of 500,000 to remove loci that were in linkage disequilibrium. This procedure retained 5807 loci that resolved population structure marginally better than the full dataset. Using the Green Bay and main basin pairwise *F*_ST_ values, we also created isolation‐by‐distance plots where distances were calculated as the nearest along‐shore distance between collection sites. We used a Mantel test in GenAlEx to test for a positive relationship between distance and *F*_*ST*._

### 2.4 Biophysical models

For the biophysical model, we used a Lagrangian particle tracking model previously developed to study the transport of larval cod (Churchill et al., 2011; Huret et al., 2007), where three‐dimensional current velocities and turbulent diffusivity were output from the application of the Finite Volume Community Ocean Model (FVCOM). A random walk scheme for spatially varying vertical diffusivity was used, including a vertical floating/sinking/swimming velocity (Gräwe, 2011; Rowe et al., 2016). Particles were designated to be either (1) neutrally buoyant or (2) have an upward vertical swimming velocity of 0.0003 m/s. We chose to use an upward vertical swimming velocity because yellow perch larvae are more likely to be collected in the upper layers of Lake Michigan (Martin et al., 2011). The Lagrangian particle tracking simulations were forced by output from FVCOM simulation of Lake Michigan‐Huron (Anderson & Schwab, 2013) incorporating exchange currents in the Straits of Mackinac. Horizontal grid resolution varied with finer resolution nearshore and in regions with complex coastlines (e.g., 100 m in the Straits of Mackinac to 2.5 km in the center of the lakes), and each horizontal grid was discretized into 20 terrain‐following (sigma) layers. Additional model details can be found in Supplementary Methods.

To generate connectivity matrices, the probability of transport from grid region *i* to grid region *f* was calculated as *N*_*if*_ /*N*_*i*_, where *N*_*if*_ is the number of particles initiated in grid region *i* that were within grid region *f* at the end of the simulation, and *N*_*i*_ is the total number of particles that were initiated in grid region *i* (Figures S2 and S3). Based on the FVCOM and particle tracking models, connectivity matrices were developed for 40 grid regions for 6 years: 2014–2019. The sensitivity of the connectivity matrices to model assumptions was evaluated by considering scenarios of (1) vertical swimming behavior and (2) horizontal diffusion. Simple behavior scenarios were tested, including passive particle movement and upward swimming. Scenarios with vertical swimming velocity were implemented by applying a deterministic vertical velocity in the vertical random walk turbulence scheme (Rowe et al., 2016), representing the combined effects of turbulence and directed swimming. Particles were initiated at the nodes of the unstructured FVCOM grid, at locations <10 m deep (number of nodes = 2246), consistent with nearshore spawning of yellow perch. Horizontal resolution was 200–600 m in nearshore areas where particles were initiated with 100 particles per node and uniformly distributed vertically through the water column. In scenarios with vertical swimming velocity set to zero, particles primarily remained distributed through the epilimnion, but some particles dispersed into the metalimnion in longer simulations. We also conducted scenarios with an upward swimming velocity (0.0003 m/s) sufficient to maintain particles within the epilimnion; this swimming speed was considerably less than the reported maximum horizontal swimming velocities of larval yellow perch of 0.03–0.046 m/s (reviewed by Höök et al., 2006), but sufficient to keep particles within the epilimnion (see Supplementary Methods for details). We assigned a horizontal diffusion coefficient of 5.6 m^2^/s based on estimates from Lake Michigan (Thupaki et al., 2013). Models were run for three estimates of pelagic larval duration (30, 40, and 50 days; the mean and 10 days on either side; Beletsky et al., 2007), two upward swimming velocities (0 and 0.0003 m/s), 6 weekly release dates ranging from late May to early July (the peak estimated yellow perch spawning period; Starzynski & Lauer, 2015), and 6 years (2014–2019) resulting in a total of 216 biophysical model simulations (Table 2).

**TABLE 2:** Parameter values used in the biophysical model for Lake Michigan yellow perch.

Parameter	Value
Years	2014, 2015, 2016, 2017, 2018, 2019
Release month (and week)	May(4), June(1), June(2), June(3), June(4), July(1)
Vertical velocity	0 m/s, 0.0003 m/s
Horizontal diffusivity	5.6 m^2^/s
Run duration (PLD)	30, 40, 50 days
Total number of simulations	216

*Note*: A total of 216 different model runs were conducted where all possible combinations of 6 years, 6 particle release weeks (spanning 3 months), 2 vertical upward swimming velocities, and 3 run durations were examined. Run duration consisted of the time period from when the particles were first released to when the model was stopped and are thus analogous to varying the pelagic larval duration (PLD).

### 2.5 Eco‐genetic models

To determine the extent to which currents could explain the empirically estimated genome‐wide levels of genetic differentiation among main basin sites, we created a spatially explicit, forward‐time agent‐based model that simulates larval dispersal among populations and, importantly, is parameterized by the lake‐wide biophysical connectivity matrices described above. The goal of this exercise was to find the connectivity matrix or set of connectivity matrices that best explain the empirical patterns of genetic differentiation among sample sites. We performed these analyses only for main basin sample sites (i.e., we excluded Green Bay) because the orders of magnitude higher *F*_*ST*_ between Green Bay sites and the rest of the lake made it challenging to identify the parameter values that best explained patterns of connectivity and gene flow among the main basin sample sites. The eco‐genetic model was adapted from Christie et al. (2017) to incorporate our sample design (Figure 1) and yellow perch life history characteristics. The model was parameterized with 40 sites (hereafter: “local populations”) from the 37 main basin grid regions to mimic the grid design employed by the biophysical model (Figure 1a). Three of those 37 grid regions had two sample collection sites that were both included in the model (Figure S3) for a total of 40 main basin local populations included in the model. Grid regions that did not contain a collection site were still included in the model to accurately model gene flow across the entire lake in multiple years. Each local population in the model was characterized by an average of 600–1200 individuals, resulting in an average metapopulation size of 36,000 perch. Every individual was randomly assigned a sex (male or female) and was characterized by 100 independent (unlinked) single‐nucleotide polymorphisms (SNPs). At the beginning of each model run, all multilocus genotypes were created in accordance with Hardy–Weinberg Equilibrium (HWE). After initializing populations, the model was characterized by the following steps: mortality, reproduction, larval dispersal, and recruitment. We assumed an average of 20% mortality per year, following estimates for both yellow perch and for many coastal marine species (Wilberg et al., 2005). This process created age‐structured populations with overlapping generations and a mean generation time of 4.8 years. During the mortality step, individuals were randomly removed from throughout the metapopulation. Within each local population, mortality was varied slightly each year using a random deviate from a normal distribution with a mean equal to the number of offspring needed for replacement and a standard deviation of 105. This process increased fluctuations in local population sizes and mimics population dynamics of perch populations (Irwin et al., 2009; Figure S4).

Because many aquatic organisms are characterized by high variance in reproductive success (Hedgecock & Pudovkin, 2011), we varied the number of offspring produced by each pair (with most pairs producing no offspring surviving to recruitment in a given year) using a gamma distribution with a shape parameter of 0.5 and a rate parameter of 0.1. Pairs were created by randomly pairing males and females within each local population without replacement. Offspring were created in strict accordance with Mendelian inheritance; at each locus, each offspring inherited one allele, chosen at random, from both parents. To simulate larval (i.e., offspring) dispersal among the 40 local populations, we used connectivity matrices from the FVCOM biophysical model (see section above). A total of 216 connectivity matrices were available (Table 2). For each model run, we first selected a single connectivity matrix and applied the selected connectivity matrix to each local population in the model to determine the number of recruits originating from each of the 40 local populations. Specifically, we used a multinomial distribution specifying all 40 populations, the number of needed offspring for a local population to return to its local carrying capacity, and the connectivity matrix describing the probability of a recruit originating from each of *i* local populations. In practice, the multinomial distribution was implemented prior to reproduction so that we knew precisely how many offspring to create in each local population, which increased computational efficiency; however, the actual dispersal of individuals occurred after reproduction.

Because there is a fair amount of uncertainty associated with the demographic, life history, and dispersal characteristics of Lake Michigan yellow perch, we tested a total of 4536 combinations of parameter values (Table 3), with each unique set of parameters replicated with 100 model runs. Thus, a grand total of 453,600 individual simulations were run on four high‐performance computing nodes (256 cores, 1024 GB memory). Each set of parameters consisted of a combination of parameters specified by the biophysical or eco‐genetic models (Table 3). Specifically, we selected one of three values for the number of years to run the eco‐genetic model (50, 100, or 200 years), one of three pelagic larval durations (30, 40, or 50 days), one of four estimates of local population sizes (600, 800, 1000, and 1200), one of two upward swimming speeds (0 and 0.0003 m/s), and for date‐specific current data, one of 6 years (2014–2019), and one of 6 release weeks (the 4th week of May, the 1st through 4th week of June, and the 1st week of July) (Table 3), which correspond with known peaks of yellow perch spawning events in Lake Michigan (Starzynski & Lauer, 2015). We also wanted to test whether varying connectivity matrices within a single simulation would improve predictive ability. Thus, we also included model runs where the connectivity matrix was replaced for each year of the eco‐genetic model. Specifically, we: (1) randomly selected (with replacement) a connectivity matrix from the first 3 release weeks across all 6 years (2014–2019) for every year in the eco‐genetic model, (2) randomly selected a connectivity matrix from the last 3 release weeks across all 6 years (2014–2019) for every year in the eco‐genetic model, and (3) randomly selected a connectivity matrix from all 6 release weeks across all 6 years (2014–2019) for every year in the eco‐genetic model (see sets 1–3 in Table 3). The first two scenarios were tested because the spawning window for yellow perch may be earlier (Scenario 1) or later (Scenario 2) than the entire 1.5‐month window, but not well characterized by a single release year. The last scenario (Scenario 3) tests whether averaging across all release weeks and years is a better predictor of connectivity than a single release week and year. Lastly, we performed a similar set of analyses where for each combination of parameters we randomly selected the release weeks (identical to Scenarios 1–3), but kept the year fixed (i.e., ran 1 year at a time). This procedure allowed us to test whether averaging connectivity matrices within and over years provided better predictive value than connectivity matrices that were developed from specific currents from shorter time periods (Table 3).

**TABLE 3:** Parameter values used in the eco‐genetic agent‐based model for Lake Michigan yellow perch, which was parameterized by connectivity matrices generated from a detailed biophysical model.

Parameter	Value
Model years	50, 100, 200
Matrix years	2014, 2015, 2016, 2017, 2018, 2019, 2014–2019
Matrix release month (and week)	Individual: May(4), June(1), June(2), June(3), June(4), July(1)
Matrix release month (and week) sets	Set 1 = [May(4), June(1), June(2)]
	Set 2 = [June(3), June(4), July (1)]
	Set 3 = [May(4) – July (1)]
Matrix vertical velocity	0 m/s, 0.0003 m/s
Matrix run duration (PLD)	30, 40, 50 days
Local populations	40
Local population sizes	600, 800, 1000, 1200
Annual mortality rate	0.2
Variance in reproductive success	Gamma (alpha = 0.5, beta = 0.1)
Metapopulation carrying capacity	40,000
Number of loci	100
Number of alleles per locus	2
Replicates	100
Total number of simulations	453,600

*Note*: Model years represent the number of years the eco‐genetic model was run through all steps (i.e., reproduction, dispersal, mortality). Matrix rows represent the connectivity matrices used for determining dispersal among local populations in the eco‐genetic model and correspond to the specific years, months, and weeks that particles were released in the biophysical model. Each of the 216 connectivity matrices was tested separately. We also tested specific sets of connectivity matrices where connectivity matrices were allowed to vary each year within the eco‐genetic model (i.e., a connectivity matrix designated within each set was randomly selected with replacement each model year prior to the reproduction step). For each unique combination of input parameters (N=4536), we ran 100 replicate simulations.

At the end of each model run, we calculated pairwise genetic differentiation (unbiased *F*_ST_; Weir & Cockerham, 1984) among all pairs of populations using all simulated individuals. These pairwise estimates were derived entirely from the eco‐genetic model (hereafter: “predictive values”) and were compared with the empirical *F*_*ST*_ values derived from genotyping the field‐collected samples (16 main basin adult collection sites; hereafter: “empirical values”). Thus, we obtained values of the exact same estimator calculated with two entirely independent approaches. Because the same estimator was used, a perfect fit between the predictive values and the empirical values would fall directly on a 1:1 line (i.e., y=x). Thus, to estimate the accuracy and precision of the predictive values we used simple linear regression and calculated the slope and correlation (measured here as the coefficient of determination; adjusted *R* ^2^) between the empirical and predictive values, where a perfect fit would have a slope and *R* ^2^ = 1 (Figure S5). To account for the possibility of nonlinear relationships, we also used Spearman's rank correlation coefficient, which returned nearly quantitatively identical results (Figure S6). To estimate the goodness of fit, we plotted the results of fitting a linear model to the predictive vs. empirical estimates for each set of parameters (averaged over 100 replicates) and plotted the mean correlation versus the mean slope across all unique combinations of parameter values. Parameter values resulting in high predictive power have correlation and slope values close to one and this approach allows us to isolate the effect of individual parameters against a background of thousands of different combinations of parameter values. For each parameter, we isolated the tested values (Table 3) that were in the top 20% of all simulations with respect to correlation and slope and calculated their relative contributions (e.g., predictive values generated with connectivity matrices from 2016 resulted in many more predictions in the top 20% of all parameter values than those generated from 2014). Using more stringent criteria (i.e., top 10% of all simulations) resulted in qualitatively similar results with more pronounced effects (i.e., larger effects of release year and week). We also combined slope and correlation estimates into a single goodness of fit metric (see SI Methods) to examine the contributions of specific weeks and years and to examine the relationship between larval connectivity (measured as the number of grid regions with particles originating from region *i*, averaged for all values of *i*), larval retention (measured as the number of particles that originated and remained in region *i*, averaged for all values of *i*), and goodness of fit. Lastly, the percent of variation explained by each parameter was estimated as the correlation between a specific predictor and the goodness of fit between predictive and empirical *F*_*ST*_. The eco‐genetic model and all downstream analyses were written in R version 4.0.2 (R Core Team, 2021).

## 3 RESULTS

### 3.1 Population genomics

All 959 individuals were sequenced, producing over 5 billion reads that resulted in an average of 5,463,100 paired‐end reads per sample. Following filtering, 927 individuals from 26 collection sites (delineated by site, year, and young‐of‐year vs. adult) were genotyped at 9302 loci (Table 1). Mean read depth of loci across individuals was 29x, and mean missingness per individual was 7.6% (Figure S1). The estimates of global genetic diversity for heterozygosity and allelic richness were *H*_*o*_ = 0.246, *H*_*e*_ = 0.243, and *A*_*r*_ = 1.248, respectively. Genetic diversity estimates were also calculated for each population (Table S2). An average of 36 loci (0.38%) were out of Hardy–Weinberg equilibrium (HWE) within each population (range = 0.01 to 0.89%), while only a single locus was out of HWE across 70% or more of collection sites (≥18/26 collection sites). Thus, we retained all loci for downstream analyses.

**FIGURE 2:**
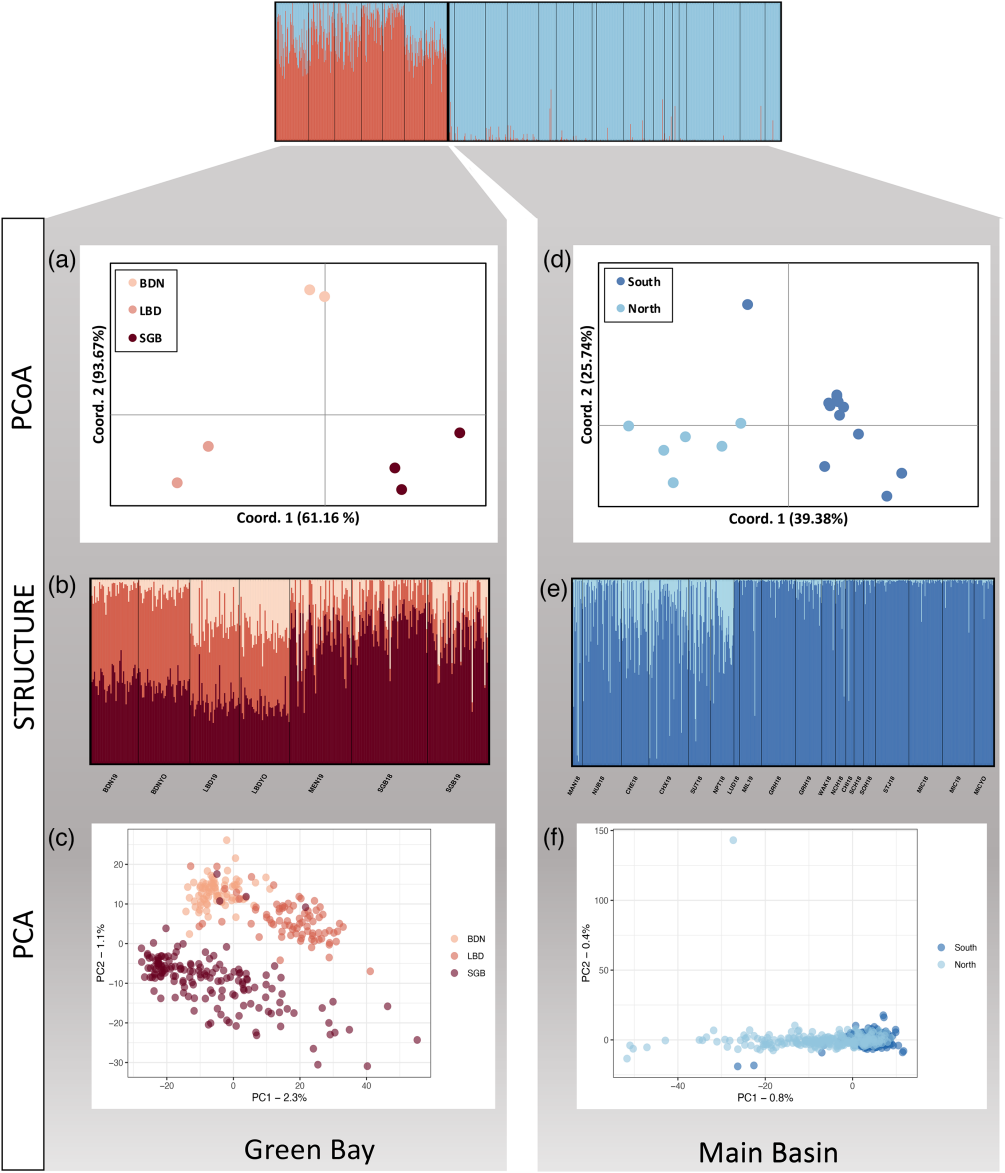
Patterns of genetic differentiation among yellow perch populations within each region of Lake Michigan. For Green Bay populations (red), principal coordinate analysis (PCoA) of pairwise *F*_*ST*_ values, where larger distances in two‐dimensional space reflect higher pairwise *F*_*ST*_ values (a), STRUCTURE output for K = 3 (b), and principal component analyses (PCA) for individual genotypes (c) all reveal genetic differentiations between Big Bay de Noc (BDN; representing two life stages: young‐of‐year (BDNYO) and adults (BDN19), Little Bay de Noc (LBD; representing two life stages: young‐of‐year (LBDYO) and adults (LBD19), and southern Green Bay (SGB; representing adults from two separate collection sites (MEN and SGB) where SGB adults were sampled in two consecutive years). For the main basin populations, there is subtle population structure between northern (MAN, NUB, CHE, CHX, SUT, and NPT) and southern Lake Michigan collection sites as again determined by principal coordinate analysis of pairwise *F*_*ST*_ values (d), STRUCTURE output for K = 2 (e), and principal component analyses for all individual genotypes (f). Collection site information and IDs can be found in Table 1.

Pairwise *F*_ST_ values ranged from −0.001 to 0.148 (Table S3) where mean pairwise *F*_ST_ was 0.003 among all main basin populations, 0.018 among all Green Bay populations, and 0.11 between the main basin and Green Bay populations. For STRUCTURE analysis across all collection sites, both mean likelihood values (L(K)) and ∆K suggested two optimal clusters (K = 2) (Figure 1; Figure S7). These results further suggest that some main basin individuals may have recent Green Bay ancestry and some Green Bay individuals have recent main basin ancestry. When visualizing higher numbers of clusters (e.g., K = 3, 4, and 8; Figure S8), we found that some of the Green Bay ancestry attributed to main basin individuals may represent subtle population structure among main basin populations. When visualizing all individuals with a PCA, we identified four main basin individuals that may have recent Green Bay ancestry (Figure 1b). Results from NewHybrids revealed that six out of 613 (1%) main basin individuals may have had recent admixture with individuals originating from Green Bay (Table S4). By contrast, 25 out of 314 (7.9%) Green Bay individuals may have had recent admixture with individuals that originated from the main basin (Table S4). The observation that a slightly higher proportion of hybrids (13%) occurred in the two sites closest to the main basin further suggests that there may be some ongoing gene flow from main basin yellow perch into Green Bay. Nevertheless, the high *F*_ST_ values and discrete clustering reveal a distinct genetic split between Green Bay populations and main basin samples (Figure 1). The principal component analysis for all individuals and principal coordinates analysis for pairwise *F*_*ST*_ values further supports this separation (Figures 1b and S9). For the PCA, there was substantially more variation among Green Bay individuals than all main basin individuals. When considering Green Bay samples on their own, STRUCTURE analysis revealed similar ∆K values for K = 2 and K = 3; however, K = 3 appears to be better supported by the results from the principal coordinate and principal component analyses (Figure 2a–c and S10). These three groupings represent Little Bay de Noc, Big Bay de Noc, and Southern Green Bay (with Menominee mostly clustering with Southern Green Bay). When the main basin samples were run separately to determine fine‐scale population structure, analysis revealed ∆K maxima at K = 2, but visualizing these clusters revealed only subtle population structure between the northern and southern sites (Figure 2d–f and S10). For Green Bay populations, there was a positive relationship between nearest along‐shore distance and *F*_*ST*_ (*p* = 0.05, *R* ^2^ = 0.29; Figure S11). For main basin populations, there was no relationship between nearest along‐shore distance and *F*_*ST*_ (*p* = 0.36, *R* ^2^ = 0.001; Figure S12) suggesting a need for additional approaches to explain patterns of genetic differentiation.

### 3.2 Biophysical and eco‐genetic agent‐based models in the main basin

**FIGURE 3:**
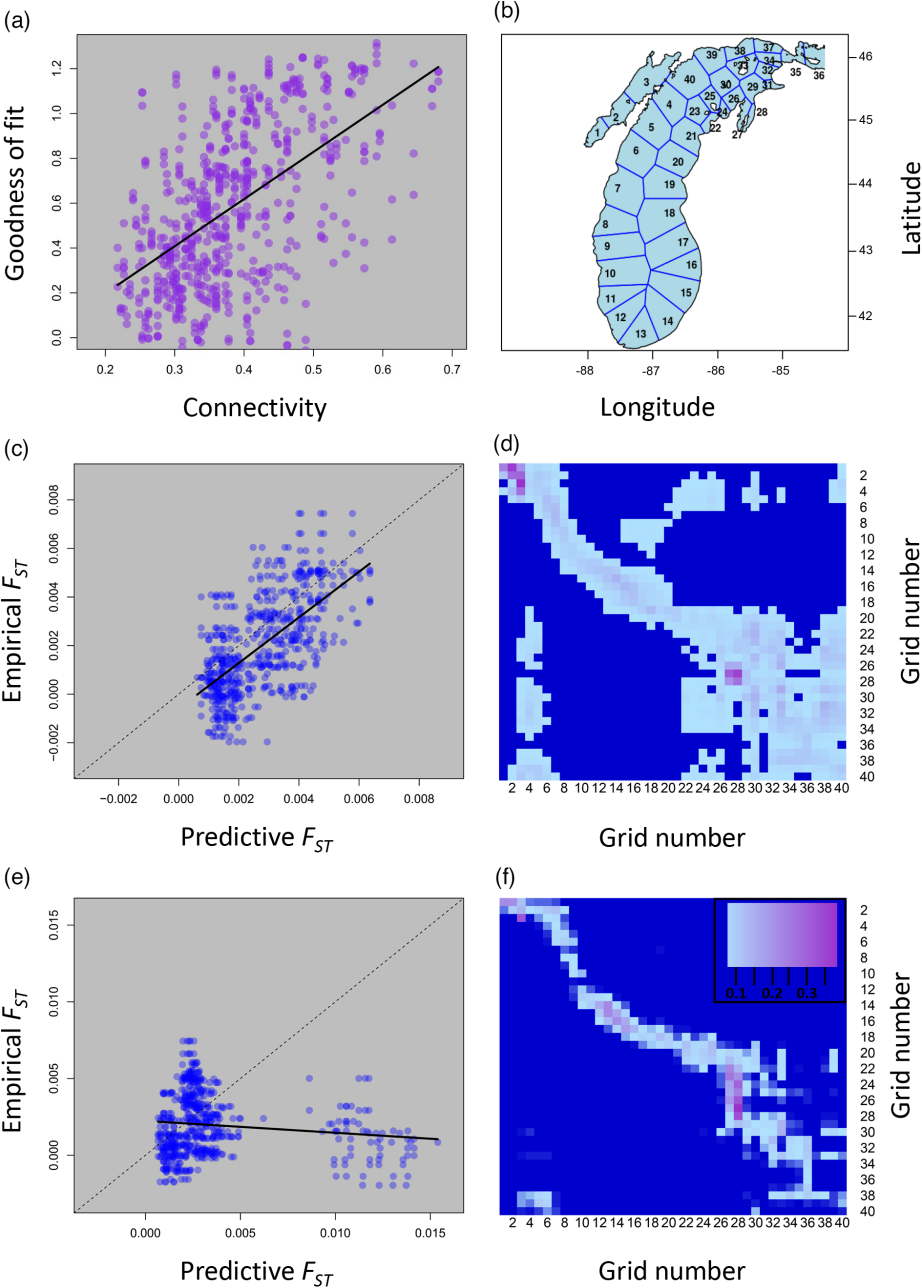
Relationships between predictive *F*_*ST*_ values among main basin Lake Michigan yellow perch populations obtained from our eco‐genetic model parameterized with biophysical current data and empirical estimates of *F*_*ST*_ from 9302 SNPs. (a) Connectivity matrices (n = 216; Table 2) derived from our biophysical model that were characterized by higher population connectivity (x‐axis) performed better at predicting (here measured as goodness of fit) our empirical estimates of *F*_*ST*_, after passing through the eco‐genetic model to generate predictive *F*_*ST*_ values, than connectivity matrices characterized by low population connectivity. (b) To track simulated particles in the biophysical models, Lake Michigan was divided into 40 roughly equally sized polygons. (c) The relationship between predictive *F*_*ST*_ and empirical *F*_*ST*_ for 10 replicated simulations for the connectivity matrix with the highest predictive ability. Each point represents a single pairwise *F*_*ST*_ comparison between two main basin sites estimated from a single simulation (x‐axis) and from RAD‐Seq data (y‐axis). A perfect fit (i.e., perfect prediction) would result in all points laying directly on the 1:1 line (dashed line). (d) The corresponding connectivity matrix with the best predictive ability (used to create predictive *F*_*ST*_ values illustrated in panel c) occurred during a time period with high connectivity among neighboring sites. (e) The relationship between predictive *F*_*ST*_ and empirical *F*_*ST*_ for 10 replicated simulations for the connectivity matrix with the lowest predictive ability. Notice that many predictive pairwise comparisons had *F*_*ST*_ values 2–3 times higher than those observed empirically. (f) The corresponding connectivity matrix, with low predictive ability, showed much lower connectivity among sites (inset illustrates color scale used in d and f where dark blue represents no connectivity between grid regions). For all scenarios, including those illustrated in panels c and e, predictive ability was assessed with 100 replicate simulations, but is illustrated here with 10 replicate simulations for visual clarity. Note that while Green Bay was included in the biophysical model (grid regions 1–3 in panel b and grids 1–3 in panels d and f), it was not included in the eco‐genetic models because the orders of magnitude higher *F*_*ST*_ made it challenging to resolve patterns among main basin populations.

For main basin populations, examining the relationship between the highest and lowest goodness of fit (i.e., slope and correlations closest to one) between the current‐derived, predictive *F*_*ST*_ values and the genotype‐derived, empirical *F*_*ST*_ values, we found that the best predictors of empirical *F*_ST_ occurred when there was high population connectivity (Figure 3a; slope = 2.10, *R* ^2^ = 0.31, *p*‐value <0.001). The single best‐fitting connectivity matrix also characterized a time period with high connectivity (Figure 3c,d). Conversely, the worst fit occurred when there was high larval retention, and low population connectivity, resulting in model‐based estimates of *F*_ST_ that were twice as high as those found empirically in the main basin (Figure 3e,f). Stated differently, when lake‐wide currents resulted in high among‐population connectivity, those matrices more accurately predicted empirical *F*_*ST*_. We also saw that lower larval retention resulted in better predictive ability, though this pattern was not as strong as connectivity (Figure S13; slope = −0.81, *R* ^2^ = 0.04, *p*‐value <0.001). When examining the relationship between connectivity and the mean current strength, we found a positive relationship (Figure S14), suggesting that periods of high connectivity were also defined by periods with stronger, more dispersive currents.

**FIGURE 4:**
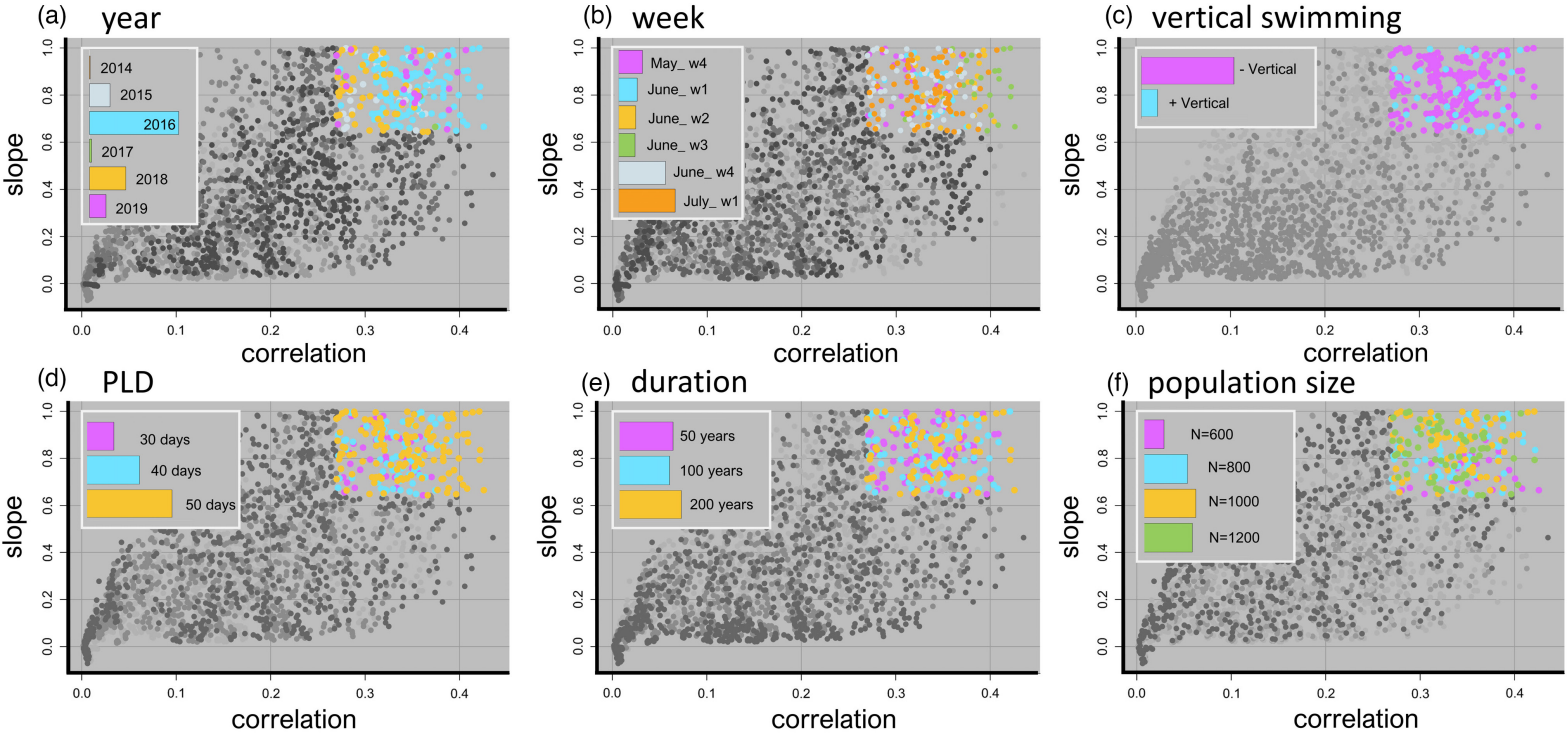
Drivers of population connectivity and genetic differentiation in main basin Lake Michigan yellow perch. Points represent the average correlation (x axis) and slope (y axis) values between predictive (generated from the integrated biophysical eco‐genetic model) and empirical *F*_*ST*_ for 100 simulations per unique set of parameter values (Table 3). A perfect fit between predictive and empirical values would lie on the 1:1 line $\left(y=x\right)$ and would have a correlation and slope equal to one. Colors represent the effect of particular parameters (across all sets of parameter values) for the top 20% of correlation and slope estimates (see Figure S15 for full color). Insets illustrate the relative contributions of particular parameter values contributing to the top 20% of model predictions. Across all parameters, the specific year and week that particles were released in the biophysical model had high predictive ability (a, b), as did vertical swimming ability (c). Pelagic larval duration, the number of years that the eco‐genetic model was run (“duration”), and the local population size had lower predictive ability (d–f).

We next found that both the year and week that larvae were released were important drivers of predictive ability (Figures 4 and S15–S18). In particular, connectivity matrices from 2016 and the last week of June and first week of July, which resulted in high levels of connectivity (Figure S18), were strong predictors of empirical estimates of *F*_ST_ (Figure 4a,b) irrespective of other parameter values. Whether or not the connectivity matrices allowed for vertical, upward swimming also had a large effect (Figure 4c), where allowing for upward vertical swimming resulted in poorer predictions. In general, eco‐genetic simulations parameterized with connectivity matrices from a 50‐day PLD, versus a 30‐day PLD had higher predictive value (Figure 4d). Varying the number of years that the eco‐genetic model was run (50 vs. 100 vs. 200 years, Table 3), and thus the number of years of reproduction, mortality, and gene flow, had little effect on the goodness of fit between predictive and empirical values (Figure 4e). Lastly, increasing the local population size for each of the 40 sites from 600 to 800 individuals improved predictive ability, but further increases in local population size had little effect (Figure 4e).

**FIGURE 5:**
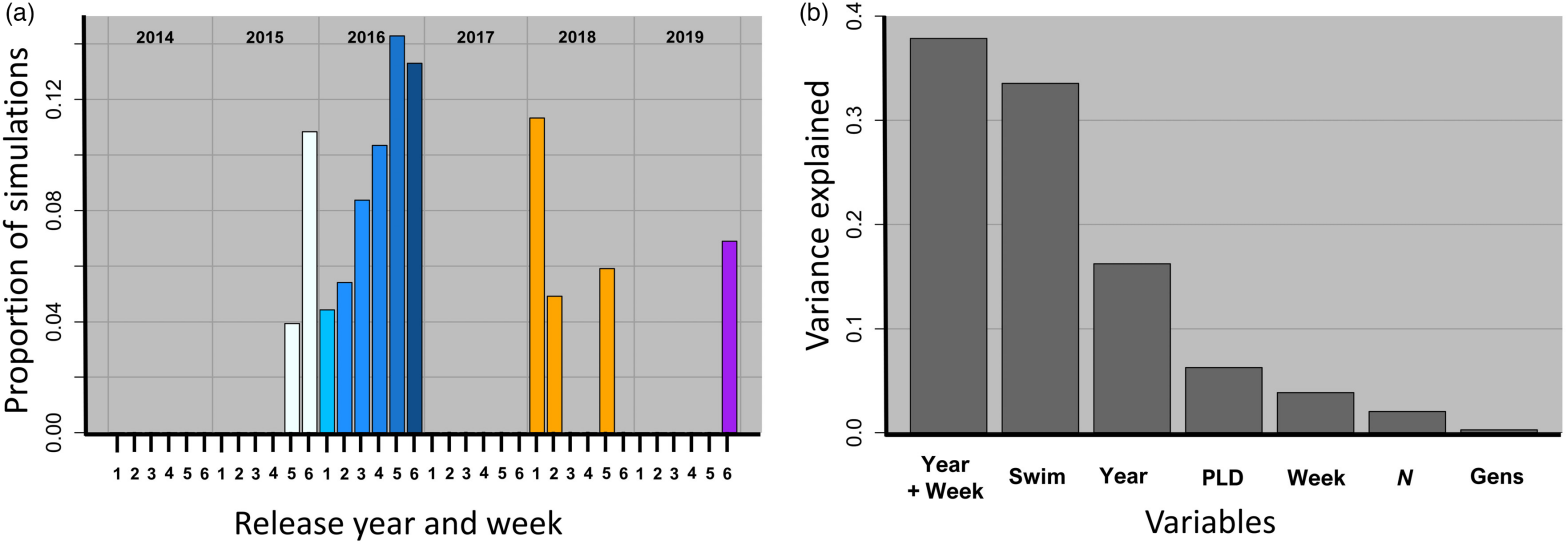
Relative contribution of dispersive currents and other drivers of genetic population connectivity in main basin yellow perch. (a) The effect of release year and week (across all sets of parameter values) for the top 20% of correlation and slope estimates illustrates that predictive values generated from connectivity matrices for specific weeks (especially those in 2016 and weeks 5 and 6) constituted a higher proportion of all integrated biophysical eco‐genetic simulations and thus have high predictive ability. The connectivity matrices with high predictive ability reflect time periods with highly dispersive currents (see Figure 3; Figure S18). Conversely, certain weeks had low predictive ability that never appeared in the top 20% of estimates (e.g., 2014). Release weeks correspond to: 1 = last week of May, 2 = 1st week of June, 3 = 2nd week of June, 4 = 3rd week of June, 5 = 4th week of June, 6 = 1st week of July. (b) The percent of variance explained by model parameters. The specific release year and week explained most of the variation, followed by vertical swimming behavior (Swim), year alone (Year), and pelagic larval duration (PLD). The week of release alone (Week), local population size (*N*), and number of generations that the eco‐genetic model was run (Gens) explained the least amount of variation.

When we examined the joint effect of release year and week, we found that the specific week of release alone had the highest predictive ability of any single parameter (Figure 5). In general, the connectivity matrices from the 4th week of June and the 1st week of July 2016 predicted the empirical estimates of genetic differentiation very well (Figure 5a, Figures S16–S18). Conversely, other weeks in different years (e.g., 2014) had very low predictive ability (Figure 5a, Figures S16–S18). This result was further confirmed with the comparatively low predictive ability of eco‐genetic output from models which were parameterized each year by randomly selected connectivity matrices across weeks and years (Figure S17). Thus, predictive *F*_*ST*_ values from models where different connectivity matrices were used in each year of the eco‐genetic simulation (i.e., analogous to averaging currents across broader windows of time) were, on average, worse at predicting empirical estimates of genetic differentiation.

When we examined the percentage of variation explained by all parameters included in the eco‐genetic model, we found that the particular week of reproduction and ensuing larval dispersal (i.e., release date) explained the majority of differences in predictive ability (Figure 5b). Vertical swimming behavior, followed by year (i.e., effect of year without respect to week), was the next best predictor (Figure 5b). The length of the pelagic larval duration, the local population size, and the number of years that the eco‐genetic model was run for explained a much smaller percentage of the variation. Very long runs of the eco‐genetic model (i.e., 1000 or 2000 years) also did not have a large effect (Figure S19). We also found that including the y‐intercept when assessing the relationship between predictive and empirical *F*_*ST*_ (and not just the slope and correlation) did not assist with model evaluation (Figure S20). Lastly, we found that the empirical pairwise main basin *F*_*ST*_ values were not influenced by outlier loci; there were very few loci with an *F*_*ST*_ value higher than 0.1 and removing the top 1% of *F*_ST_ values within each pairwise comparison had a minimal effect on the pairwise *F*_ST_ values (Figure S21).

## 4 DISCUSSION

For an aquatic species with a pelagic larval stage, we demonstrated that genetic differentiation across a 500 km latitudinal gradient is best predicted by strong and highly dispersive currents. Our results suggest that abiotic and biotic factors that increase dispersal distances (e.g., limited upward swimming, spawning at certain times of year) will favor population connectivity and contribute to observed population structure. More generally, the observation that genetic differentiation in large aquatic systems may best be determined by strong and highly dispersive currents has broad implications. First, many examinations of population connectivity rely solely on data from biophysical models and often average or integrate current data over lengthy periods of time (Selkoe et al., 2010). Here, we show that such analyses could be problematic because the realized population genetic connectivity may be best characterized by a small subset of possible connectivity matrices. Thus, parameterizing connectivity matrices for use in theoretical or demographic models of population connectivity must be performed with caution. Second, the joint observations that (1) model runs employing multiple connectivity matrices (randomly selected from multiple weeks and years; Figure S17) performed worse than those from single release dates, and (2) the number of years for which the eco‐genetic model was run had almost no effect on predictive ability (e.g., Figures 4e and S19) suggest that even 6 years of biophysical data may not be sufficient to fully characterize patterns of gene flow over evolutionary time scales. This result contrasts somewhat with recent studies (e.g., Legrand et al., 2022), which found that multigenerational coalescent models had high predictive value. Nevertheless, it was somewhat surprising to us that connectivity matrices based on current patterns from a single release date had higher predictive ability than sets of all the connectivity matrices that captured all or a subset of the variability in the 6 years by varying connectivity matrices each year. This result is most parsimoniously explained by the possibility that the 6 years for which detailed biophysical data were available do not fully capture the variability found over evolutionary timescales. Thus, over longer time scales, it is more likely that the connectivity matrices were more similar to the individual matrices that were found to be good predictors of the empirical patterns of genetic differentiation and thus were characterized by strong currents. Although it is not statistically improbable that certain weeks would have higher predictive ability than others given the high interweek variation in currents, it is highly unlikely that those same weeks would also happen be the weeks with the strongest currents (and highest connectivity; Figures 3e and S18). Lastly, we speculate that in highly dynamic systems, such as many marine and freshwater environments, if most population connectivity is driven by a small subset of possible connectivity matrices, then this result could explain, at least in part, many observations of chaotic genetic patchiness (Broquet et al., 2013; Johnson & Black, 1982).

Bolstering our interpretation that strong and dispersive currents play a disproportionate role in determining patterns of population connectivity is the observation that 2015 and 2016 were particularly strong recruitment years for yellow perch in the main basin of Lake Michigan (Makauskas & Clapp, 2018). In turn, these year classes dominated the 2018 and 2019 perch population in the main basin (Makauskas & Clapp, 2018). Annual recruitment success of yellow perch in Lake Michigan and the entire Great Lakes region is positively related to spring–summer temperatures (Honsey et al., 2016). Of interest, 2016 (the year with best predictive ability) was particularly warm and 2014 (a year with very low predictive ability) was particularly cold. Annual differences in temperature also influence when yellow perch spawn and hatch. For example, Withers et al. (2015) documented earlier peak catches of recently hatched larval yellow perch in southern Lake Michigan during 2010, a particularly warm year, than during 2011, an intermediate thermal year. Whether highly dispersive currents facilitate not only genetic population connectivity, but also recruitment, remains unknown as does the interaction between temperature, currents, and recruitment. This question could potentially be answered by an even sampling of different age classes, especially young‐of‐year; however, we found it particularly challenging to collect younger age classes from the main basin due to their low abundance. The exact mechanisms by which climatic conditions lead to strong year classes are unknown. With water currents in Lake Michigan being primarily wind and temperature driven, it is likely that climate conditions that favor strong recruitment events also favor specific water current patterns (although it is unclear to what extent the realized water current patterns drive recruitment success). We illustrate here that there are specific water current patterns (including periods of highly dispersive currents) that are consistent with observed connectivity patterns. These influential connectivity patterns may have been observed repeatedly over time and overlapped with periods of strong recruitment success. Nevertheless, more work is needed to explore variation in yellow perch spawn timing, population connectivity, and subsequent recruitment.

Here, we integrated biophysical and eco‐genetic, agent‐based modeling to better understand the drivers of population connectivity in aquatic systems. This approach could be improved with higher‐resolution oceanographic data, both in space and time. At least some of the unexplained variation between model‐derived and empirical estimates of genetic differentiation could be due to a lack of resolution in the biophysical model, particularly with respect to nearshore currents (Gawarkiewicz et al., 2007; Swearer et al., 2019). Furthermore, accurate estimates of local population sizes combined with accurate estimates of fecundity could improve both the biophysical model, in terms of more accurately determining the number of particles to release at each site, and the eco‐genetic model, in terms of accurately simulating gene flow and genetic drift. Releasing larger numbers of larvae per node and incorporating additional aspects of larval behavior such as horizontal swimming, particularly as yellow perch are strong swimmers toward the end of their PLD, could also help to improve the biophysical model (Kingsford et al., 2002; Leis & McCormick, 2002). An alternative approach would be to simulate de novo (i.e., without using the biophysical model) a wide range of connectivity matrices and determine which set had the best predictive ability; however, the de novo creation of connectivity matrices may generate connectivity matrices that have very high predictive power, but that were unlikely to ever occur in reality. Likewise, estimating larval mortality, settlement competency, and settlement probability relative to habitat availability could yield additional improvements. Additional data on spawning times and locations (i.e., yellow perch may spawn in southern Lake Michigan earlier than yellow perch in northern Lake Michigan) may also bolster the ability to explain empirical patterns of genetic differentiation. Improving the eco‐genetic model to incorporate site‐specific demography and adult movement data, along with additional evolutionary forces such as mutation and selection, could also yield improvements. Moreover, whole‐genome sequencing may allow for more accurate estimates of genetic differentiation, more precise demographic reconstruction, and the identification of putatively adaptive loci that could explain regional responses to selection in different environments. In short, any approaches that remove uncertainty in parameter estimates are likely to yield gains in prediction accuracy and aid in disentangling the relative contributions for drivers of population connectivity. Lastly, there may be better approaches for deciding which eco‐genetic models have the highest predictive ability. Nevertheless, distilling model evaluation into two parameters (slope and correlation) does have its advantages. For example, in Figure 4, we found that some predictive estimates do have slopes that are close to 1, meaning that some models are not systematically over or underestimating gene flow or genetic drift. However, the maximum correlation estimates illustrated in Figure 4 only approach 0.5, which suggests that there is either room for improvements to the models themselves or that there is some inherent stochasticity in high gene flow systems that may never be fully predicted.

At a regional level, we found that Green Bay was highly genetically differentiated from the main basin (mean *F*_ST_ = 0.11), which further highlights the importance of managing Green Bay and main basin yellow perch as distinct stocks. This confirmation of previous work by Miller (2003) is most likely driven by the relatively low rates of water exchanged between Green Bay and the main basin (Beletsky & Schwab, 2001), but other factors such as differences in reproductive traits (e.g., spawn timing) or local adaptation (e.g., differential selection after dispersal) may be further contributing to reduced gene flow between Green Bay and the main basin. Tagging studies suggest that there is limited movement of adult yellow perch between Green Bay and the main basin. Glover et al. (2008) tagged 63,948 yellow perch in Green Bay and the main basin and recaptured 3801 of these tagged fish. Of these recaptured fish, they found 0 individuals that moved between Green Bay and main basin. This study and others demonstrate that adult yellow perch generally do not move very far and is again suggestive that spatial connectivity is primarily driven by larval transport. Other studies have also suggested that limited water exchange is a barrier to gene flow between Green Bay and the main basin (e.g., Miller, 2003) and measurements and simulation models also demonstrate limited water exchange. Our results from NewHybrids suggest that there are greater levels of gene flow into Green Bay from the main basin than gene flow out of Green Bay into the main basin (Table S4). If this result is correct, it may be driven either by larval dispersal or by adult main basin yellow perch migrating into Green Bay—a pattern sometimes seen in drowned river mouth lakes (Chorak et al., 2019). Another possibility is that moderate genetic divergence among Green Bay collection sites is increasing the “hybridization rate” within Green Bay sites themselves. Within Green Bay itself, moderate spatial population structure was evident (*F*_ST_ = 0.018). Thus, for management purposes it may be appropriate to consider separate management strategies for perch from different regions of Green Bay (e.g., northern Green Bay versus southern green Bay). We also documented subtle population structure within the main basin itself (Figure 2d,e) where northern and southern sites tend to cluster apart from each other. The genetic differences within the main basin, however, are much more comparable to marine systems, with mean pairwise *F*_*ST*_ equal to 0.003. Of note, yellow perch populations from Traverse Bay were much more similar to main basin yellow perch populations than Green Bay yellow perch were to main basin populations. This result suggests that the local, and perhaps fine‐scale, currents within Traverse Bay result in more connectivity with the main basin of Lake Michigan than is currently appreciated. From a population genetic perspective, it is interesting that Green Bay yellow perch show characteristics in common with fishes that lack a pelagic larval stage (higher *F*_ST_; isolation‐by‐distance; Figure S11), while main basin perch show patterns of genetic differentiation similar to many marine fishes (lower *F*_*ST*_; no pattern of isolation‐by‐distance Figure S12) (Martinez et al., 2018). Our three young‐of‐year samples each showed patterns of ancestry similar to their nearby adult samples (Figure 2, most easily seen in STRUCTURE plots), suggesting that high variance in reproductive success is not driving patterns of genetic differentiation in this system (cf. Christie, Johnson, et al., 2010, Christie, Tissot, et al., 2010), but more samples of fish across different age classes are needed.

In conclusion, we found that populations of an ecologically and commercially important fish with a 40‐day pelagic larval stage are connected by highly dispersive currents. Identifying the characteristics of currents that drive population connectivity can allow for better conservation and management decisions such as identifying the appropriate size and spacing of protected areas (Baetscher et al., 2019; Carr et al., 2017) or determining the boundaries of populations and management units (Waples & Gaggiotti, 2006). Spatial components of the main basin Lake Michigan yellow perch stock have clearly been overharvested in the past (Wilberg et al., 2005). However, indices of overharvest (e.g., skewed sex ratios, altered maturation schedules) recovered rapidly with relaxation of fishing pressure, potentially due to mixing of yellow perch from other regions of the main basin (Feiner et al., 2015; Lauer et al., 2008). Thus, maintaining such connectivity is likely important for the resiliency of the overall stock. Moreover, knowing which connectivity matrices have the best predictive ability has utility for researchers and managers. For example, if researchers would like to model metapopulation dynamics to attempt to predict patterns of recruitment, understand where to best place protected areas, or model responses to fisheries‐induced evolution, they now know which set of connectivity matrices best predicts the observed empirical patterns of genetic differentiation among populations. Future studies may further explore annual variation in source–sink dynamics of yellow perch in Lake Michigan to determine which locations may be particularly important to protect as contributors of recruits. Scaling up these analyses to multiple species within a community could not only allow for greater generalizations, but could also identify the drivers, and their relative contributions, of population and community connectivity within and among aquatic systems.

## CONFLICT OF INTEREST STATEMENT

The authors declare no conflicts of interest.

## Supporting information


Data S1.

## Data Availability

All RAD‐Seq data are available at the NCBI as BioProject ID PRJNA902284 and are available at http://www.ncbi.nlm.nih.gov/bioproject/902284. All filtering, population structure analyses, and eco‐genetic modeling code are hosted at GitHub: https://github.com/ChristieLab/yellow_perch_dispersal and all biophysical data are available at Dryad: https://doi.org/10.5061/dryad.ns1rn8pzr.
