# Supplementary material for: Dispersive currents explain patterns of population connectivity in an ecologically and economically important fish
Source: Evol Appl. 2023 Jun 21;16(7):1284–301. doi: 10.1111/eva.13567 (PMC10363847; doi:10.1111/eva.13567)
Supplement: Supplementary file 1 — Data S1. [file EVA-16-1284-s001.docx]

**Supplementary Materials for:** "Dispersive currents explain patterns of population connectivity in an ecologically and economically important fish"

Disclaimer: Any use of trade, product, or firm names is for descriptive purposes only and does not imply endorsement by the U.S. Government

Claire Schraidt, Amanda S. Ackiss, Wesley A. Larson, Mark D. Rowe, Tomas O. Höök, Mark R. Christie

**Supplementary Methods:**

*Biophysical models*

The finite volume community ocean model (FVCOM) is an unstructured grid, finite-volume, free surface, three-dimensional primitive equation ocean model that solves the momentum, continuity, temperature, salinity, and density equations (Chen, Liu, and Beardsley 2003). The unstructured grid of FVCOM conforms to complex coastline morphologies and allows for increased grid resolution in regions of interest. Turbulence closure was implemented through the MY-2.5 scheme for vertical mixing (Galperin et al. 1988), and the Smagorinsky scheme for horizontal mixing (Smagorinsky 1963). FVCOM has been previously implemented for the Great Lakes yielding accurate predictions of temperature, water levels, and currents (Anderson et al. 2015; Anderson, Schwab, and Lang 2010; Anderson and Schwab 2013; Bai et al. 2013). Skill assessment of the model showed improved simulation of currents and surface temperature in comparison to the previous generation of Great Lakes operational forecast models, and it is currently used as NOAA’s next-generation Great Lakes Operational Forecast System. We define the epilimnion as the upper layer, or surface-mixed-layer, of a thermally stratified lake, which was an outcome of the vertical turbulent diffusivity simulated by FVCOM and the metalimnion as the layer below the epilimnion, separating the epilimnion from the hypolimnion, characterized by a strong temperature gradient and low turbulent diffusivity.

*Additional goodness of fit measures*

To provide an additional estimate the goodness of fit, we first standardized estimates of the slope to have a maximum value of 1 because many values were greater than 1 (*i.e.*, slope estimates of 0.8 and 1.2 were standardized to a value of 0.8 as they are equally distant from the 1:1 line and the direction of deviation does not matter for goodness of fit). We next added the standardized slope and *R^2^* values together assuming an additive relationship and that a perfect fit between the estimated and predictive value would have a slope of 1 and an *R^2^* of 1 (all points would fall on the 1:1 line). Thus, our goodness of fit values could range from < 0 (there were a few negative slopes) to 2 (perfect fit). For each combination of parameter values and release dates, we calculated the average predictive ability (goodness of fit averaged over 100 simulations) of the eco-genetic model at explaining the empirical values. We also examined the relationship between larval connectivity (measured as the number of grid cells with particles originating from cell *i*, averaged for all values of *i*), larval retention (measured as the number of particles that originated and remained in cell *i*, averaged for all values of *i*), and goodness of fit.

**References**

Lombardy. 2015. “Reconstruction of a Meteotsunami in Lake Erie on 27 May 2012: Roles of Atmospheric Conditions on Hydrodynamic Response in Enclosed Basins.” *Journal of Geophysical Research, C: Oceans* 120 (12): 8020–38.

Anderson, Eric J., and David J. Schwab. 2013. “Predicting the Oscillating Bi-Directional Exchange Flow in the Straits of Mackinac.” *Journal of Great Lakes Research* 39 (4): 663–71.

Anderson, Eric J., David J. Schwab, and Gregory A. Lang. 2010. “Real-Time Hydraulic and Hydrodynamic Model of the St. Clair River, Lake St. Clair, Detroit River System.” *Journal of Hydraulic Engineering* 136 (8): 507–18.

Bai, Xuezhi, Jia Wang, David J. Schwab, Yi Yang, Lin Luo, George A. Leshkevich, and Songzhi Liu. 2013. “Modeling 1993–2008 Climatology of Seasonal General Circulation and Thermal Structure in the Great Lakes Using FVCOM.” *Ocean Modelling* 65 (May): 40–63.

Chen, Changsheng, Hedong Liu, and Robert C. Beardsley. 2003. “An Unstructured Grid, Finite-Volume, Three-Dimensional, Primitive Equations Ocean Model: Application to Coastal Ocean and Estuaries.” *Journal of Atmospheric and Oceanic Technology* 20 (1): 159–86.

Galperin, B., L. H. Kantha, S. Hassid, and A. Rosati. 1988. “A Quasi-Equilibrium Turbulent Energy Model for Geophysical Flows.” *Journal of the Atmospheric Sciences* 45 (1). <https://www.researchgate.net/profile/B_Galperin/publication/234840522_A_Quasi-equilibrium_Turbulent_Energy_Model_for_Geophysical_Flows/links/0fcfd5101bd39c2244000000/A-Quasi-equilibrium-Turbulent-Energy-Model-for-Geophysical-Flows.pdf>.

Luu, K., Bazin, E. and Blum, M.G., 2017. pcadapt: an R package to perform genome scans for selection based on principal component analysis. *Molecular ecology resources*, *17*(1): 67-77.

Smagorinsky, J. 1963. “General Circulation Experiments with the Primitive Equations: I. The Basic Experiment.” *Monthly Weather Review* 91 (3): 99–164.





**Figure S1**: Percent of genotypes retained (1 - proportion missing) per locus (a) and per individual (b) after filtering. The average amount of missing data per locus was 7.60% across all individuals. The average amount of missing data per individual across all loci was 6.48%. The average % missing values are similar due to the filtering options used but notice that the variation differs.


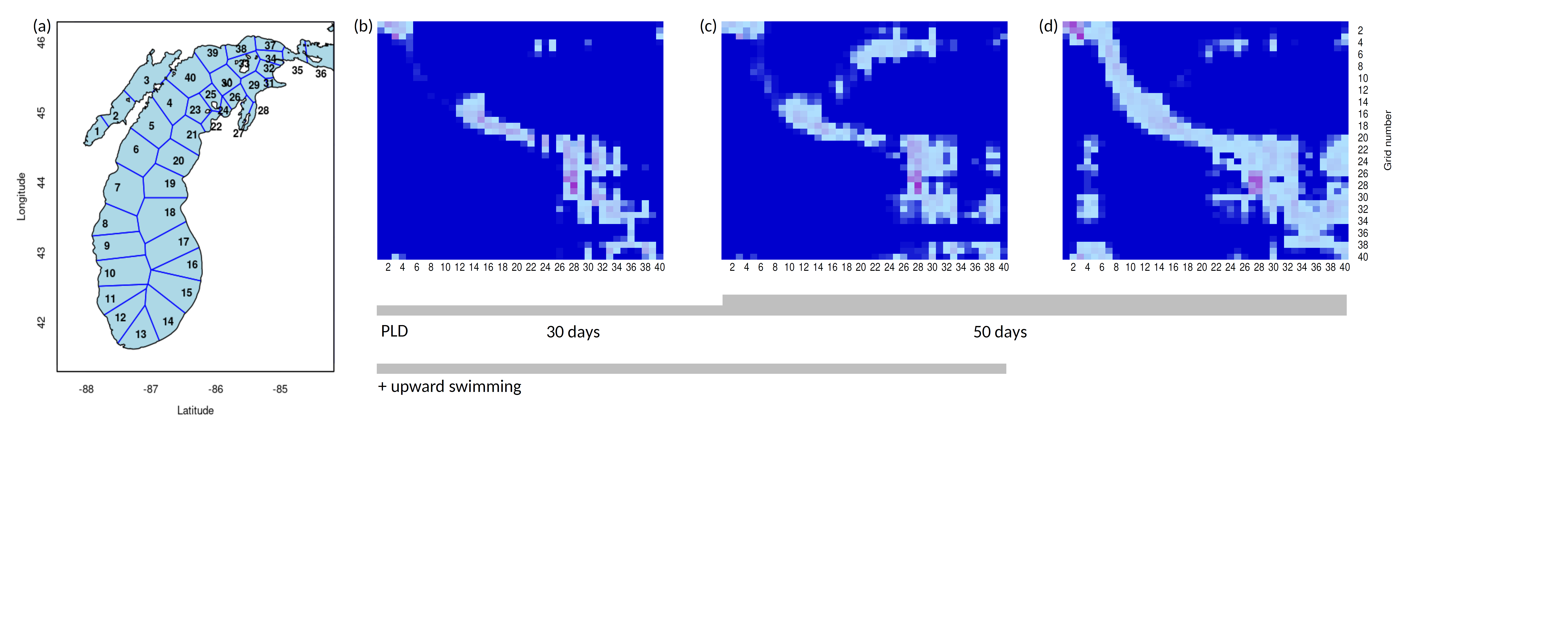


**Figure S2:** Current-driven patterns of Lake Michigan population connectivity as identified via biophysical models. (a) In order to track simulated particles, Lake Michigan was divided into 40 roughly equally sized polygons. Connectivity matrices from particles released June 30, 2019 illustrate population connectivity for particles with a simulated 30-day pelagic larval duration (PLD) and with upward swimming (0.0003 m/s) (b), particles with a simulated 50-day pelagic larval duration and upward swimming (c), and particles with a 50-day pelagic larval duration and no upward swimming (d). In these examples, connectivity increases with increases in the pelagic larval duration and neutral buoyancy (no upward swimming). Notice that there is 1.) high population connectivity within Green Bay, 2.) more population connectivity within northern than southern main basin sites, and 3.) high larval retention and a general pattern of northward dispersal in the main basin.


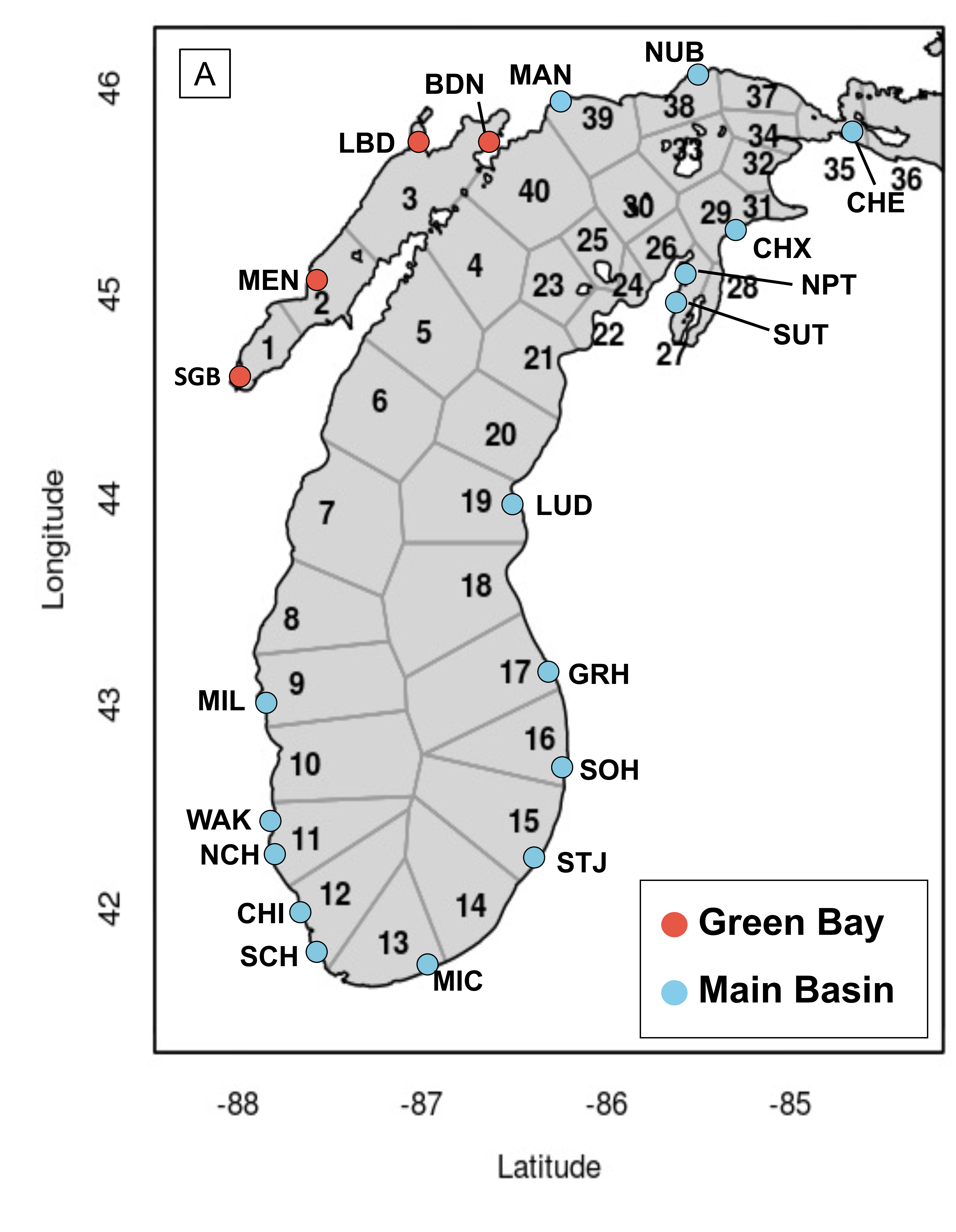


**Figure S3:** Sample collection sites with the grid regions from the biophysical model superimposed on tip. A total of 960 yellow perch (*Perca flavescens*) were collected and genotyped from 20 sites, representing 26 collections (Table 1), circumscribing Lake Michigan. To track simulated particles, Lake Michigan was divided into 40 roughly equally sized polygons (numbers 1-40). Three grid regions had 2 sample collection sites that were both included in the model. Grid regions that did not contain a collection site were still included in the model to accurately model gene flow across the entire lake in multiple years (i.e., 40 local populations were included in the eco-genetic model – one for each of the 37 main basin grid regions plus additional populations for the grid regions that contained two sample sites; grid regions 11, 12, and 27).


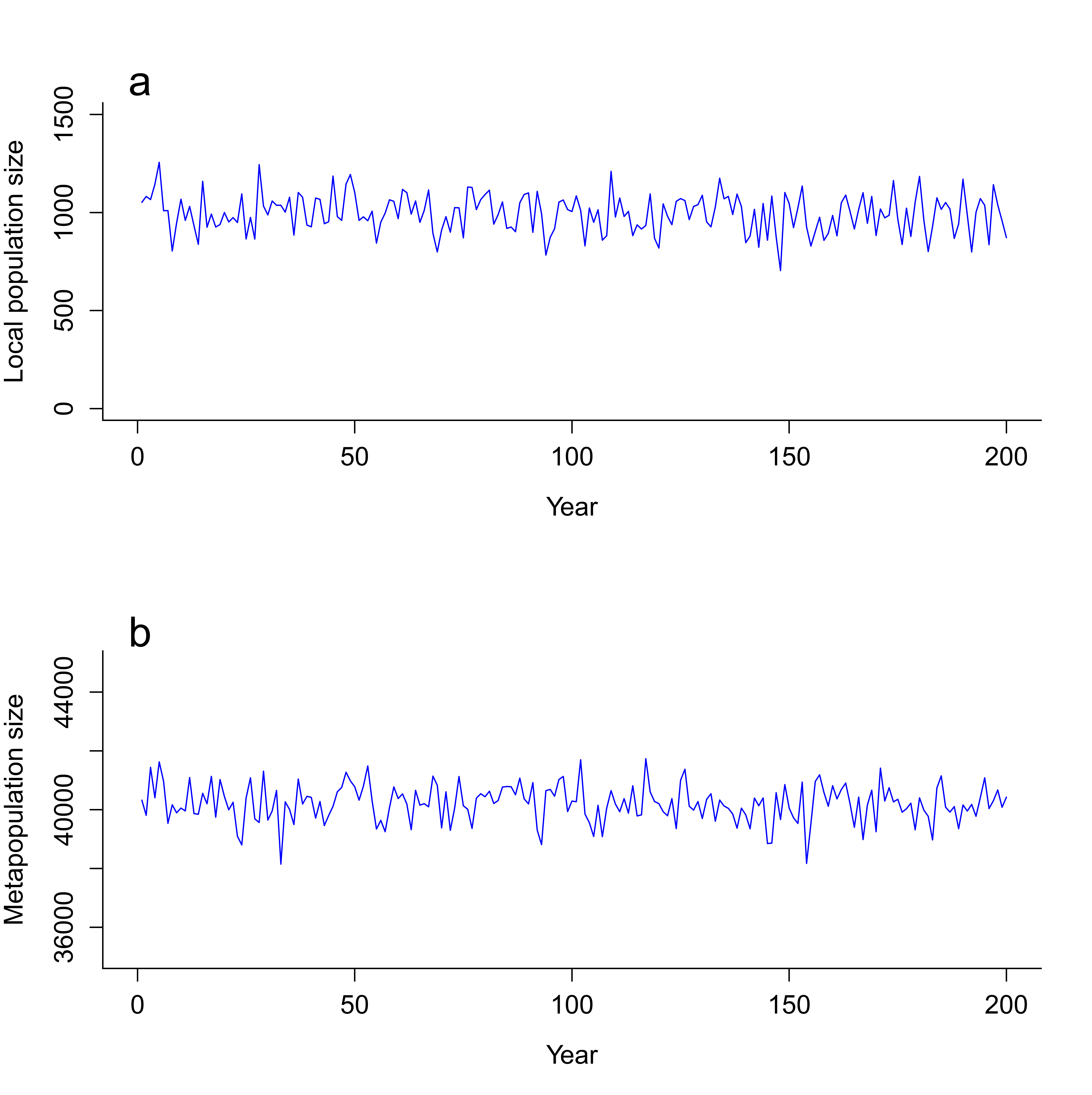


**Figure S4:** Examples of population dynamics through time in (a) one of 40 modeled local populations and (b) across all 40 local populations (*i.e*., the metapopulation). Notice that there was variation in each local population per year that, when summed over all local populations, resulted in fluctuations for the entire metapopulation. This process mimics population dynamics of Great Lakes perch populations


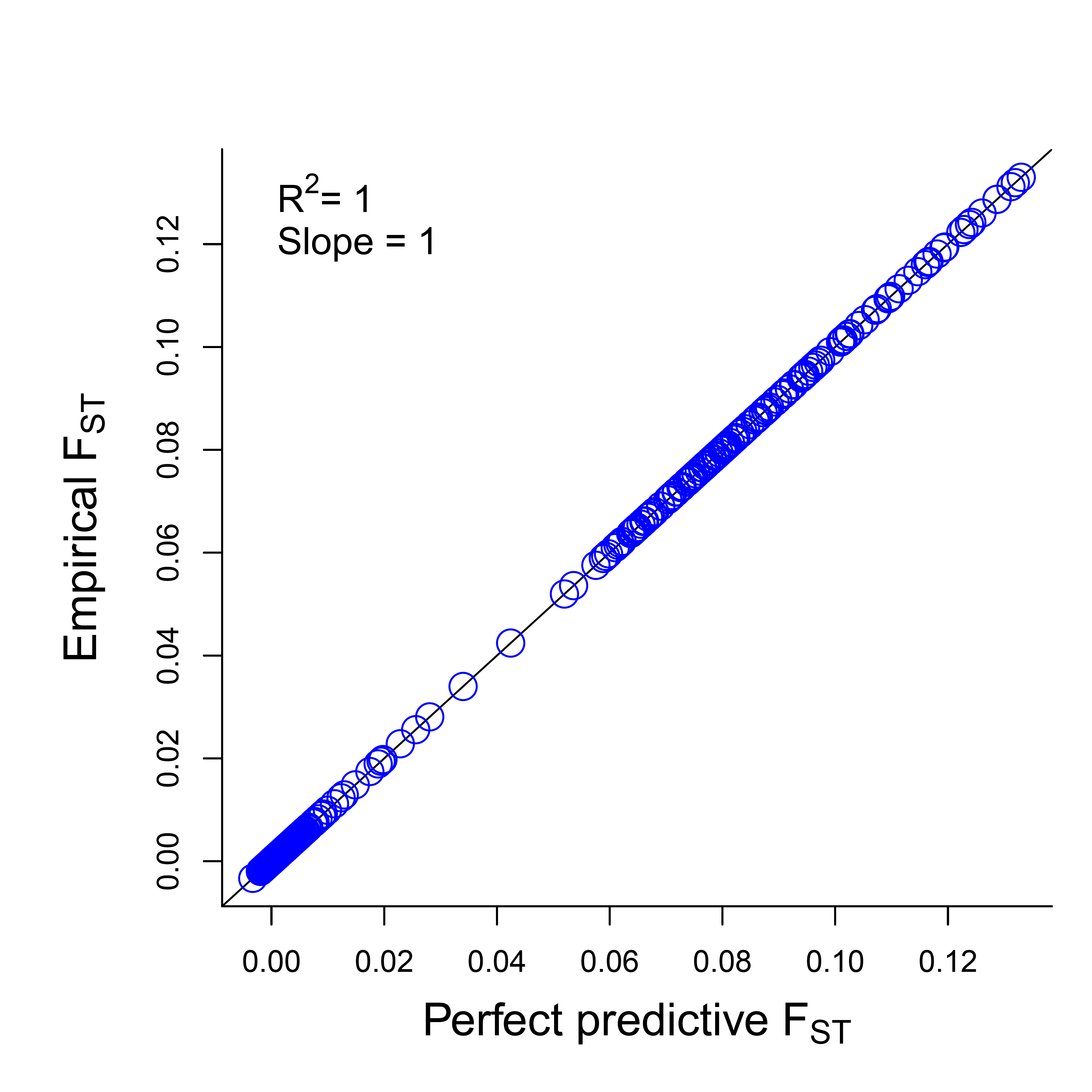


**Figure S5:** Relationship between predictive values of *F_ST_* that would perfectly predict empirical estimates. Here we simply plotted all empirical *F_ST_* values against themselves, illustrating a hypothetical example of perfect prediction. Notice that the slope and *R*^2^ values both equal 1 and the points fall directly on the 1:1 line ($y=x$). In reality, perfect prediction is unlikely to occur due to measurement errors in *F_ST_* and models that are unlikely to ever capture all the variation present in natural systems. However, models that more accurately predict empirical estimates should produce predictive values resulting in slopes and correlations that are closer to 1 (see Figure 3 as an example).


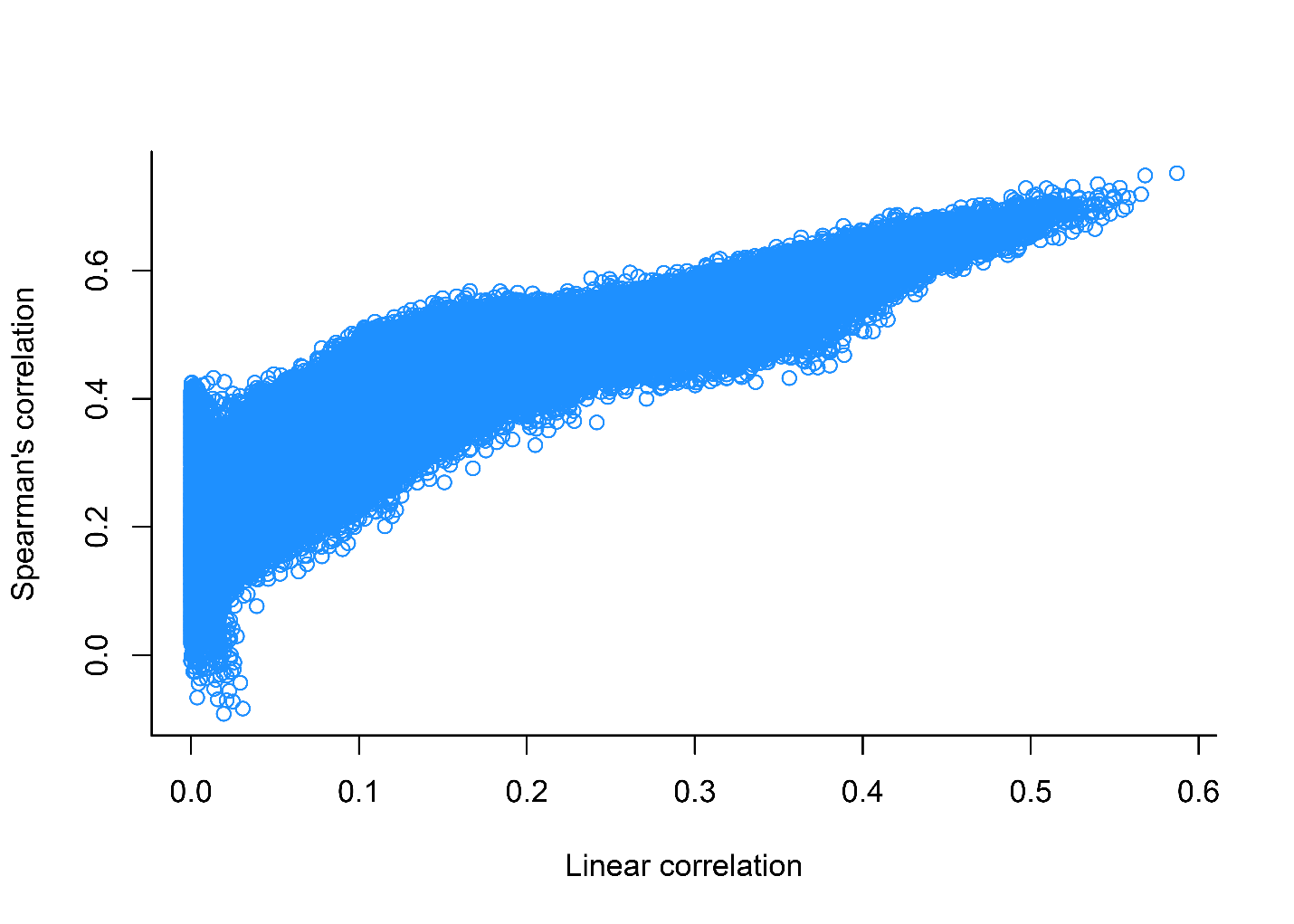
**Figure S6:** Relationship between linear correlation and Spearman’s rank correlation for estimating goodness of fit. The results were largely similar such that we relied on linear correlation estimates for the remainder of the analyses.





**Figure S7:** Changes in K values for all collection sites using STRUCTURE suggesting that no improvements were gained for larger numbers of clusters (see main text for details) and that it is sufficient to highlight the two main clusters illustrated in Figure 1 (but see Figure S8 for plots with additional K values).


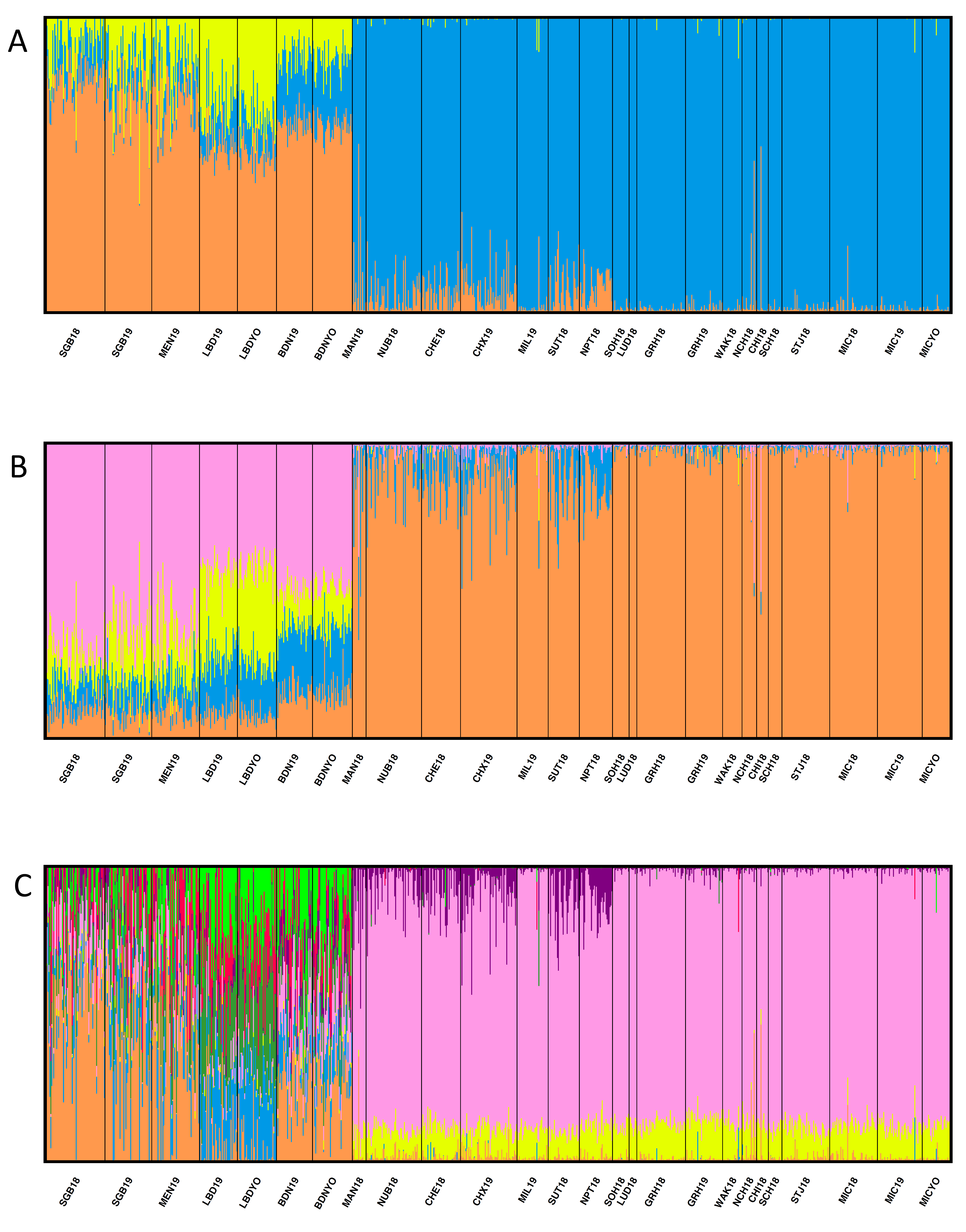


**Figure S8:** Visualization of STRUCTURE results for K= 3 (A), K= 4 (B), and K=8 (C). K=2 is best supported (see Figure S7). Note that there are still a handful of main basin individuals that appear to have Green Bay ancestry with increasing K, however the majority of main basin individuals with “Green Bay ancestry” when K=2 appear to be individuals characterized by the subtle population structure found among northern and southern main-basin populations (see Figure 2). This interpretation is further confirmed with the PCA of all individuals (Figure 1).


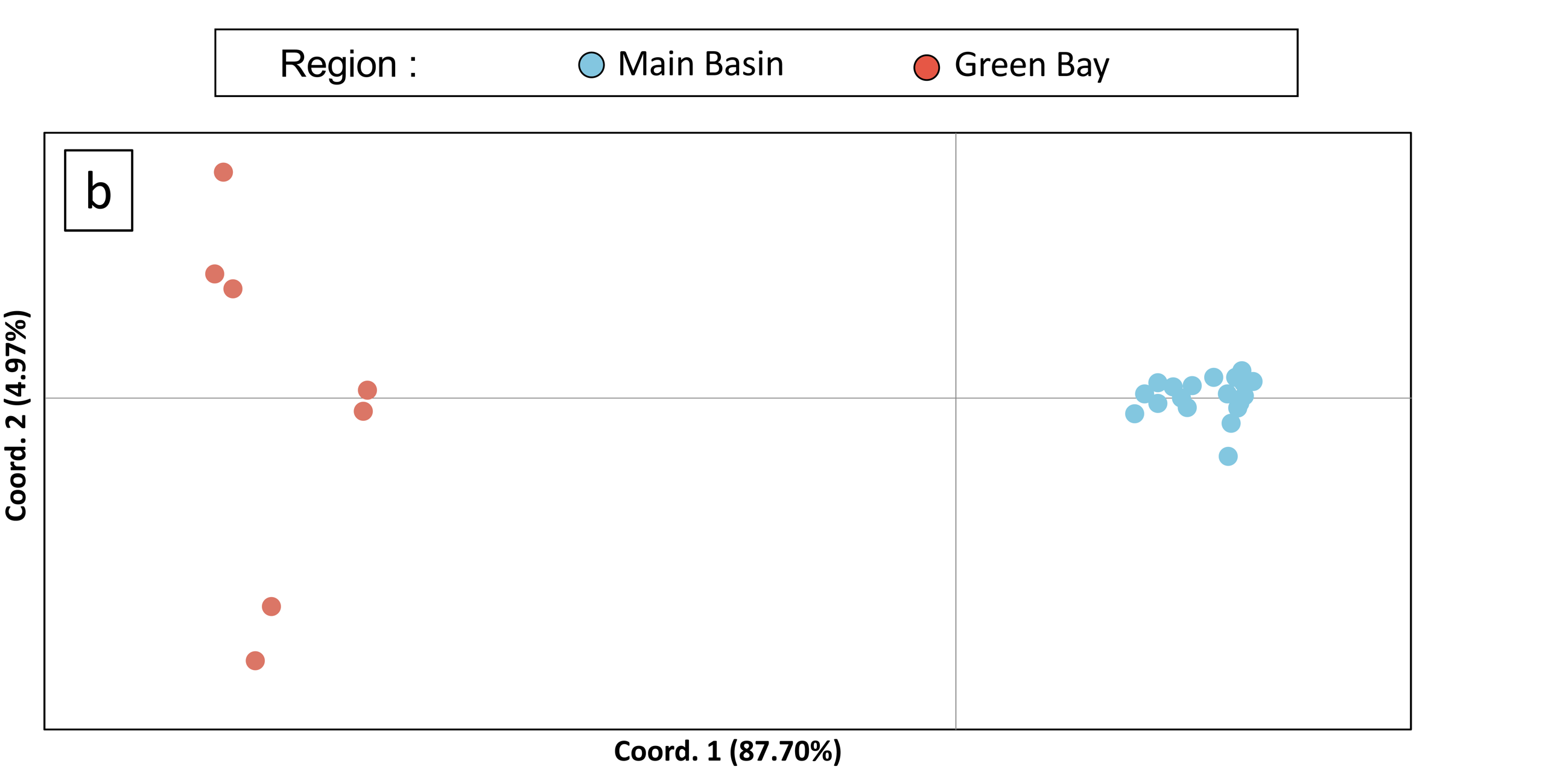


**Figure S9:** Principal coordinate analysis (PCoA) of all pairwise *F_ST_* values for all 26 collections (representing 20 geographic sites), where larger distances in two-dimensional space reflect higher pairwise *F_ST_* values. See Table 1 for sample details and Table S3 for pairwise *F_ST_* values. Notice the distinct separation between collection sites from Green Bay and the main basin along the x-axis (Coordinate 1), which explains more than 87% of the variation.





**Figure S10:** Changes in K values for all Green Bay (left panel) and all main basin (right panel) analyzed separately with STRUCTURE. In Green Bay, STRUCTURE analysis revealed similar ∆K values for K = 2 and K = 3, however K = 3 appears to be better supported by the results from the principal coordinate and principal component analyses (Figure 2a,b,c). These three groupings represent Little Bay de Noc, Big Bay de Noc, and Southern Green Bay, respectively. When the main basin samples were run separately to determine fine-scale population structure, analysis revealed ∆K maxima at K=2, but visualizing these clusters revealed only subtle population structure between the northern and southern sites (Figure 2d,e,f)


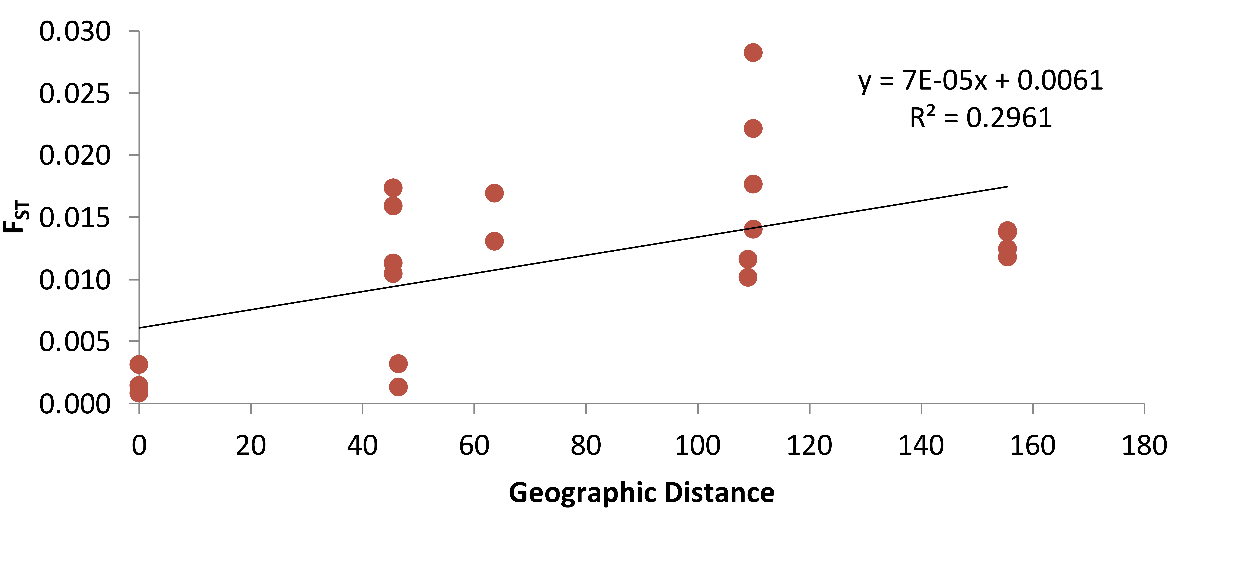


**Figure S11:** Isolation by Distance relationship for Green Bay collection sites. Plotted is the nearest along-shore distance (kilometers) versus pair-wise unbiased *F_ST_*. Mantel tests revealed that the slope is different than one (p < 0.05). Figure 2 also reveals differences among northern and southern Green Bay collection sites.


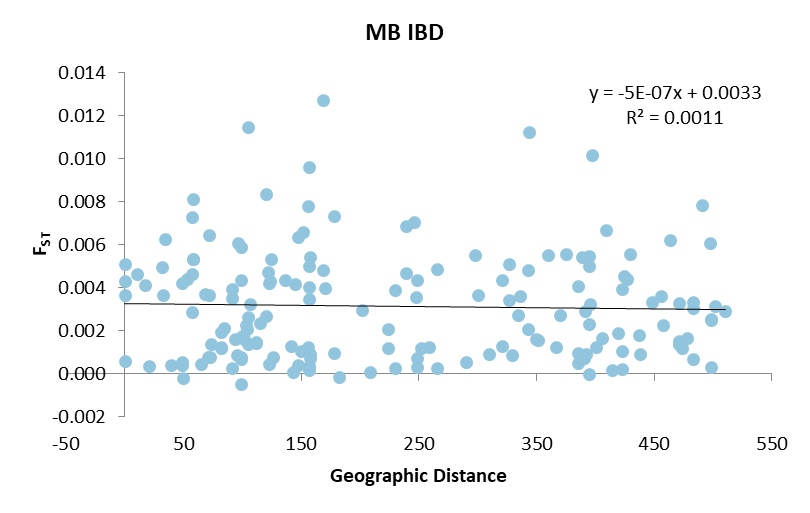


**Figure S12:** Isolation by Distance relationship for all main basin collection sites. Plotted is the nearest along-shore distance (kilometers) versus pair-wise unbiased *F_ST_*. Mantel tests revealed that the slope is not different than one (p = 0.36), suggesting that there is no relationship between along shore distance and genetic differentiation for main basin Lake Michigan yellow perch populations.


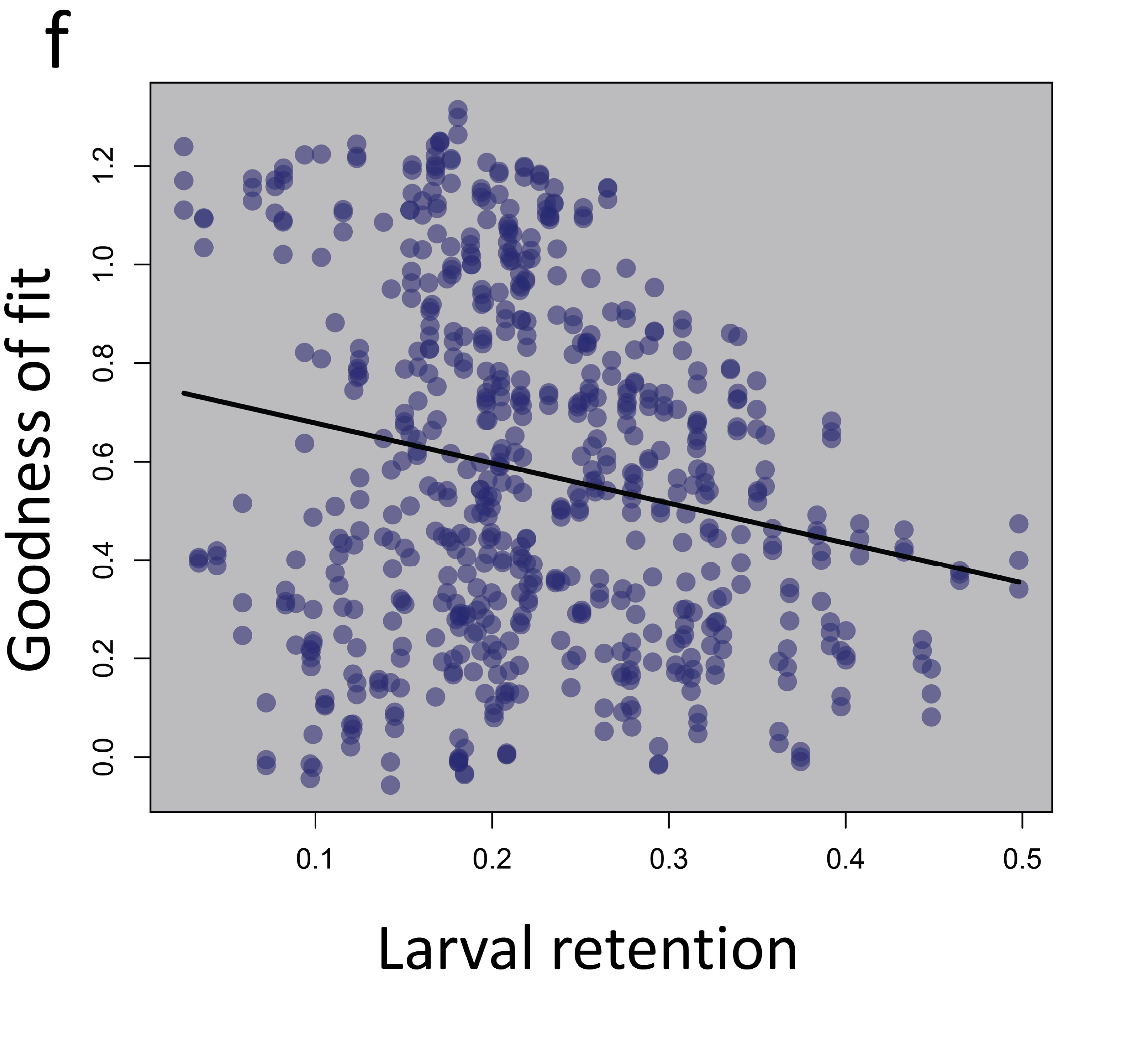


**Figure S13:** In general, connectivity matrices with higher population connectivity had a higher predictive ability than matrices with low population connectivity (Figure 3). Similarly, connectivity matrices with higher rates of larval retention (figure above) had a lower predictive ability (slope = -0.81, *R^2^* = 0.04, p-value < 0.001), though this relationship was not as strong as for connectivity.


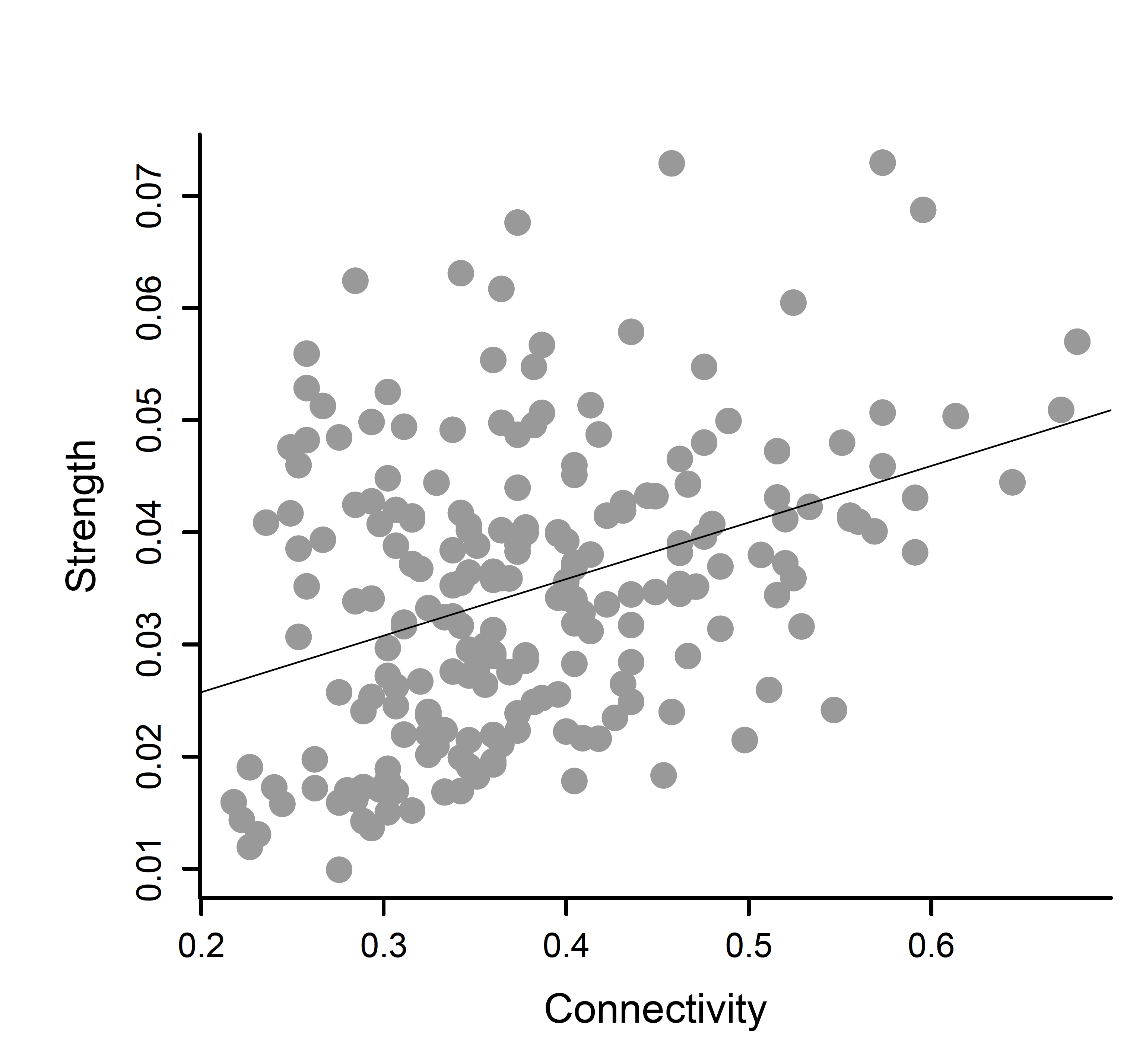


**Figure S14:** Figure illustrating the relationship between connectivity and strength of the currents for all connectivity matrices generated from the biophysical model. We measured “strength” as the standardized mean number of particles that moved between grid regions (standardized by the total number grid regions that exchanged at least 1 particle) (*R^2^* = 0.13, *p* < 0.001).





**Figure S15:** Full color version of Figure 4. Drivers of population connectivity and genetic differentiation in main basin Lake Michigan yellow perch. Points represent the average correlation and slope values between predictive (generated from the integrated biophysical eco-genetic model) and empirical *F_ST_* for 100 simulations per unique set of parameter values (Table 3). A perfect fit would between predictive and empirical values would lie on the 1:1 line $(y=x)$ and would have a correlation and slope equal to one. Colors represent the effect of particular parameters (across all sets of parameter values). Insets illustrate the relative contributions of particular parameter values contributing to the top 20% of model predictions. Across all parameters, the specific year and week that particles were released in the biophysical model had high predictive ability (a, b), as did vertical swimming ability (c). Pelagic larval duration, the number of years that the eco-genetic model was run (“duration”), and the local population size had lower predictive ability (d, e, f).


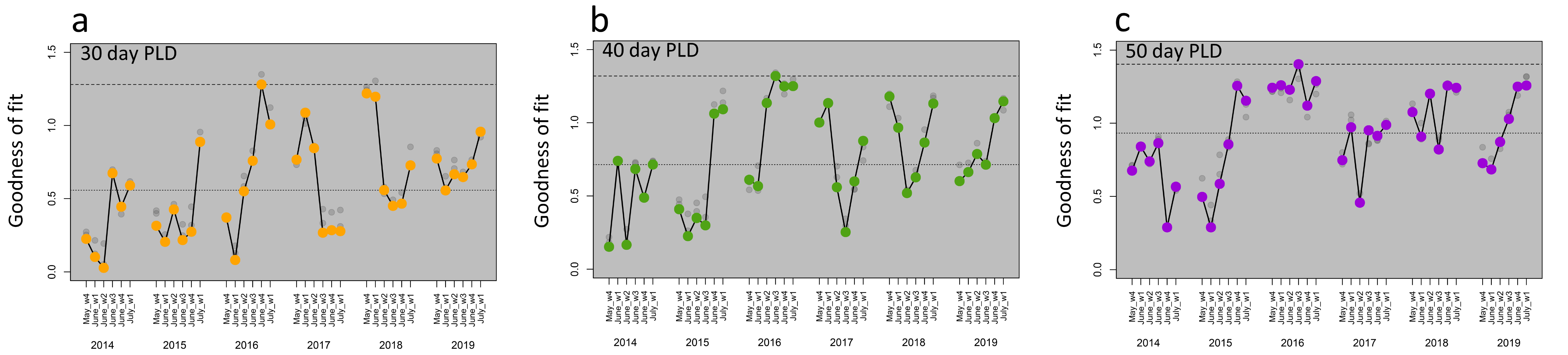


**Figure S16:** Drivers of population connectivity and genetic differentiation in Lake Michigan yellow perch. Points represent the average goodness-of-fit values between predictive and empirical *F_ST_* for 100 simulations per set of parameter values and are separated by particle release year and week. Colors represent pelagic larval durations of (a) 30 days (orange points), (b) 40 days (green points), and (c) 50 days (purple points) where particles were neutrally buoyant. The median and best goodness of fit values are illustrated with dashed, horizontal lines. For each combination of parameters, the eco-genetic model was run for 50, 100, or 200 years with the highest average goodness of fit indicated with the colored points (200 years in panels a-c) and the other two years (i.e., 50 and 100) indicated with smaller grey points. In some cases, the grey points lie directly under the colored point and are not visible. The specific release year and week explained the largest percentage of variation among all parameters examined. In this figure, goodness of fit was measured as described in Supplementary Methods.


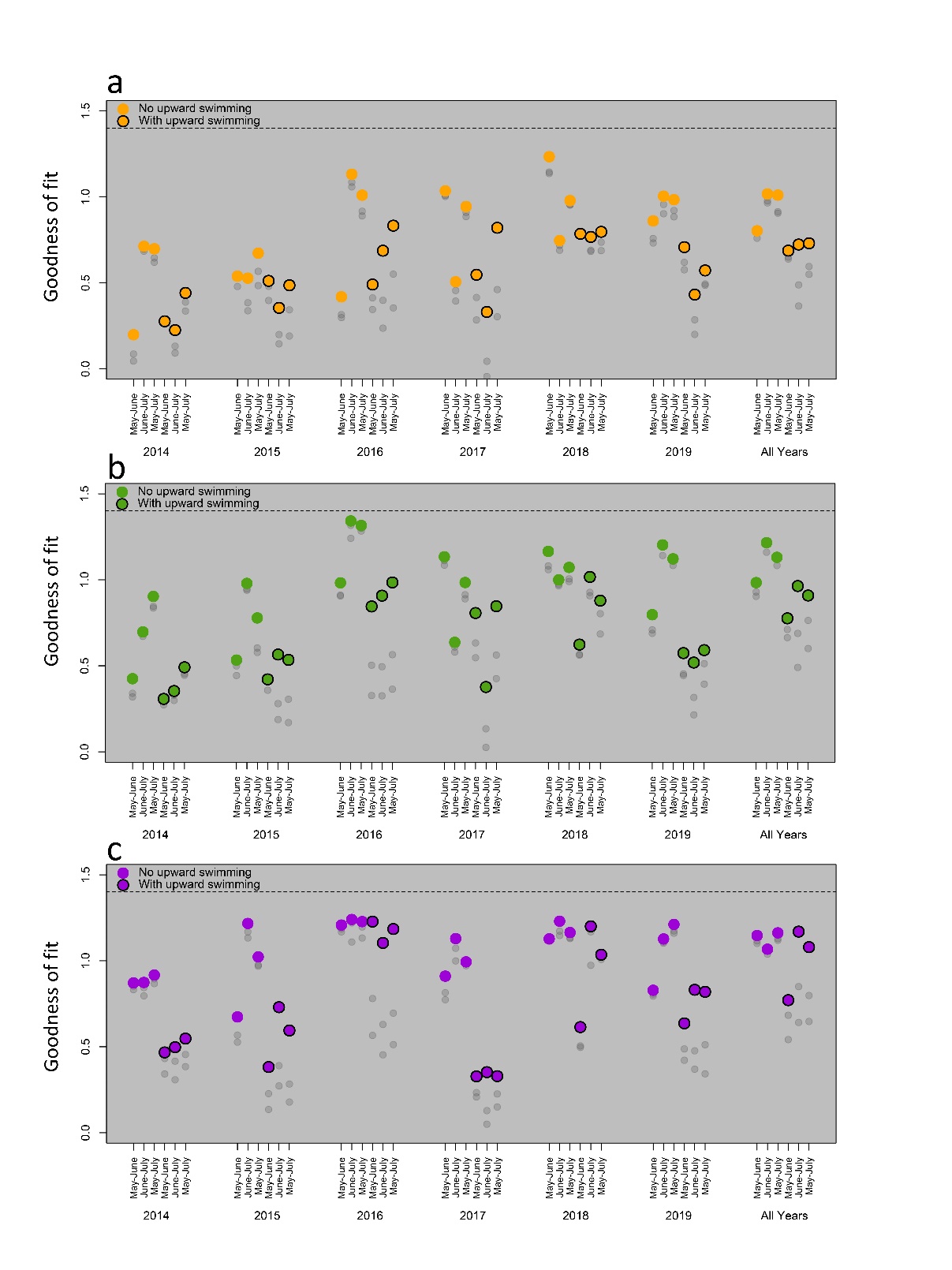


**Figure S17:** Goodness of fit between predictive *F_ST_* derived from our biophysical-ecogenetic model and empirical *F_ST_*. Here we evaluated whether varying connectivity matrices within a single eco-genetic simulation would improve predictive ability. We examined model runs where we: 1.) randomly selected a connectivity matrix from the first 3 release weeks for each year in the eco-genetic model ("May-June"; for each year of the biophysical model 2014-2019) , 2.) randomly selected a connectivity matrix for the last 3 releases for each year in the eco-genetic model ("June-July"), and 3.) randomly selected a connectivity matrix from all 6 release weeks to be used for each year in the eco-genetic model ("May-June"). Lastly, we performed a similar analysis where for each combination of parameters and for each single release date, we randomly selected the year (2014-2019) and the release week from which drew the connectivity matrix ("All Years"). The horizontal dashed line represents the best goodness of fit value from Figure S16.


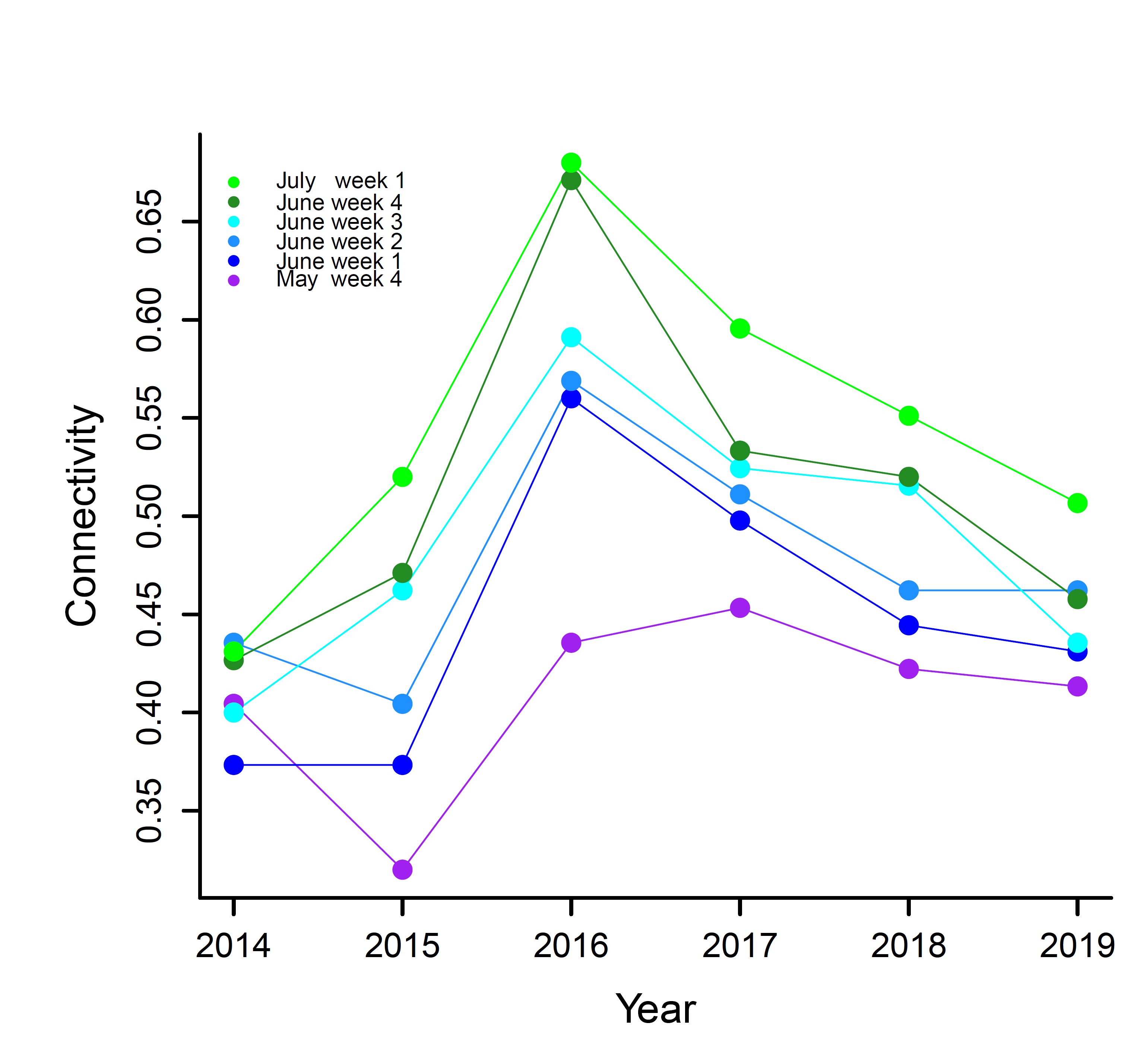


**Figure S18:** Relationship between release year and week and connectivity as estimated directly from the connectivity matrices from the biophysical model. Notice that the last week in June and first week in July in 2016 had particularly high connectivity. These same two weeks also generated the best predictive values of *F_ST_* after being run through the eco-genetic agent-based model (See Figures 3-5). Conversely, 2014 had low connectivity and also generated *F_ST_* values with low predictive ability.


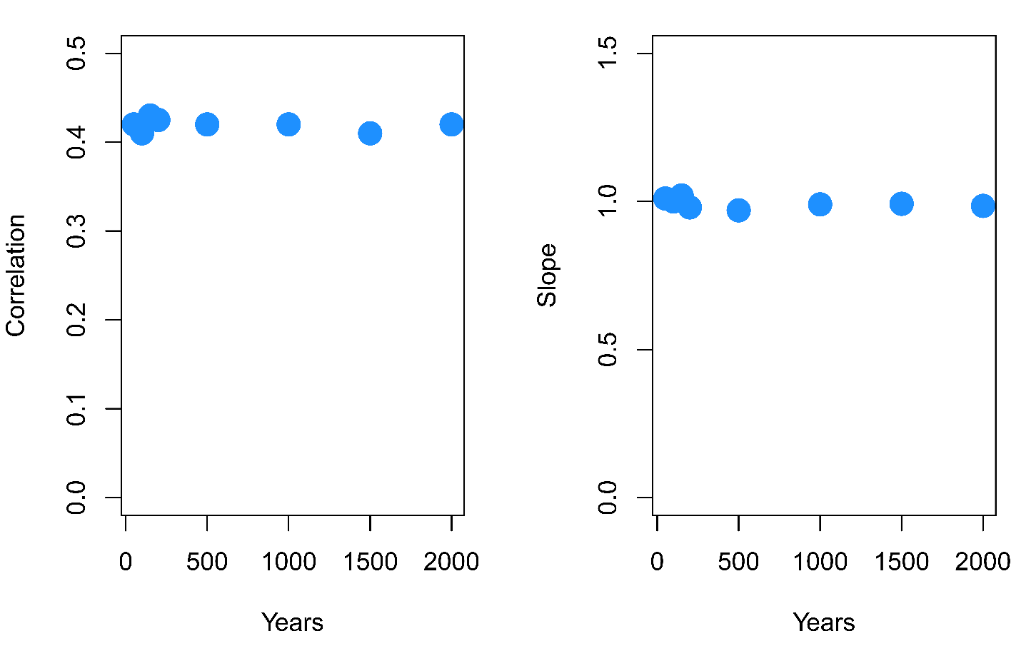


**Figure S19:** Examining the effect of long runs of the eco-genetic model on predictive ability. Here we picked one of the scenarios with highest predictive ability (week 5, 2016) and varied the number of years that the model was run, testing 50, 100, 150, 200, 500, 1000, and 2000 years). A total of 100 replicate simulations were run for each unique combination of parameters. Plotted are the mean correlation and slope for estimates of predictive estimates of *F_ST_* regressed against empirical estimates of *F_ST_*. Notice that varying the number of years that the eco-genetic model is run for has negligible effect on predictive ability (see also Figure 4 and Figure 5).


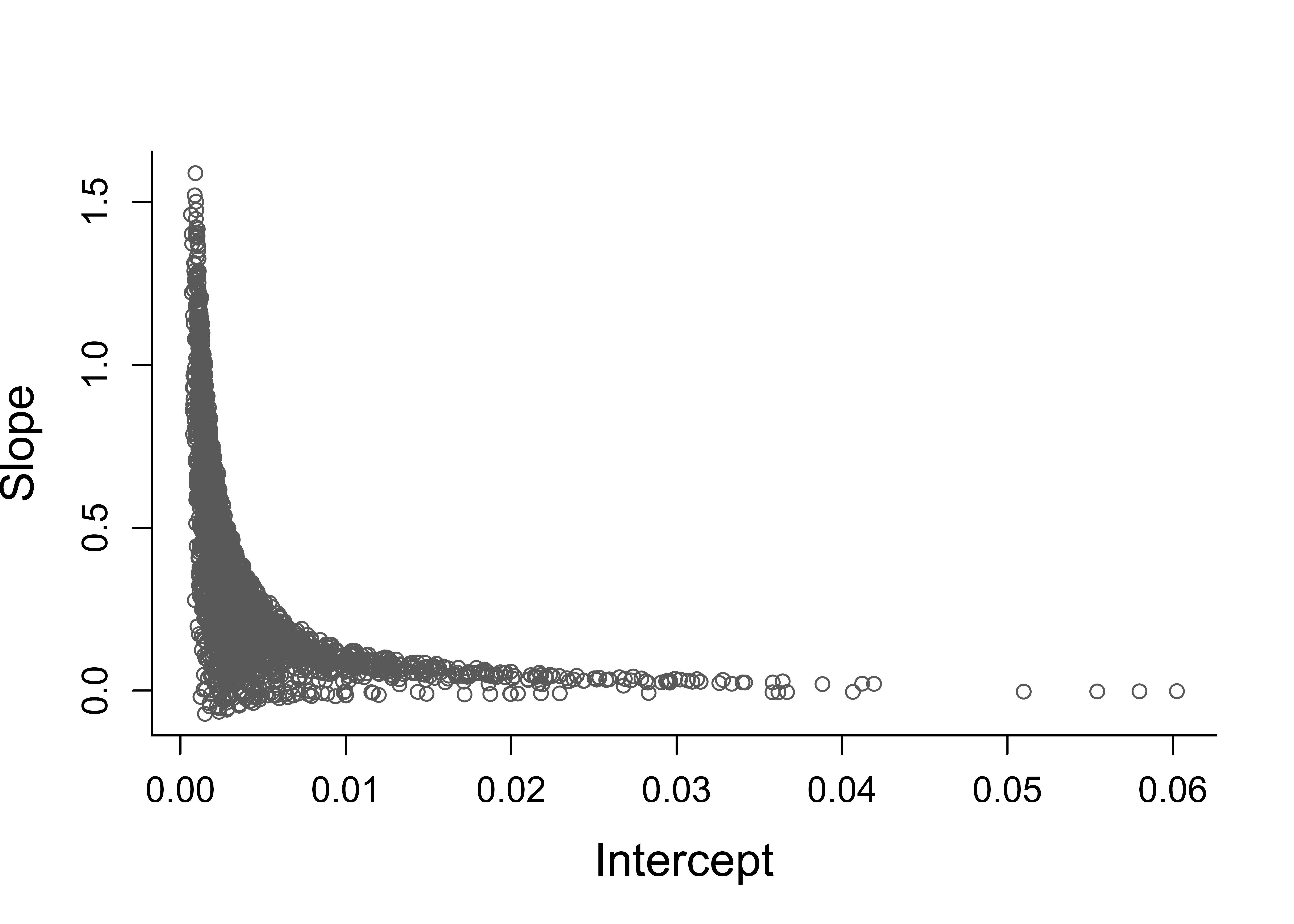


**Figure S20:** Relationship between the intercept and slope for all of our model runs where we fit a linear model between predictive and empirical *F_ST_*. A perfect fit between predictive and empirical data would lie exactly on the y=x line, resulting in a slope of 1, a correlation of 1, and an intercept of 0. Notice that when the intercept was far from 0, the slope was always close to 0, whereas when the slow was close to 1 (indicating a good fit), the intercept was always close to 0. Thus, using information about the intercept would not improve the evaluation and assessment of the parameter space for our specific set of models.


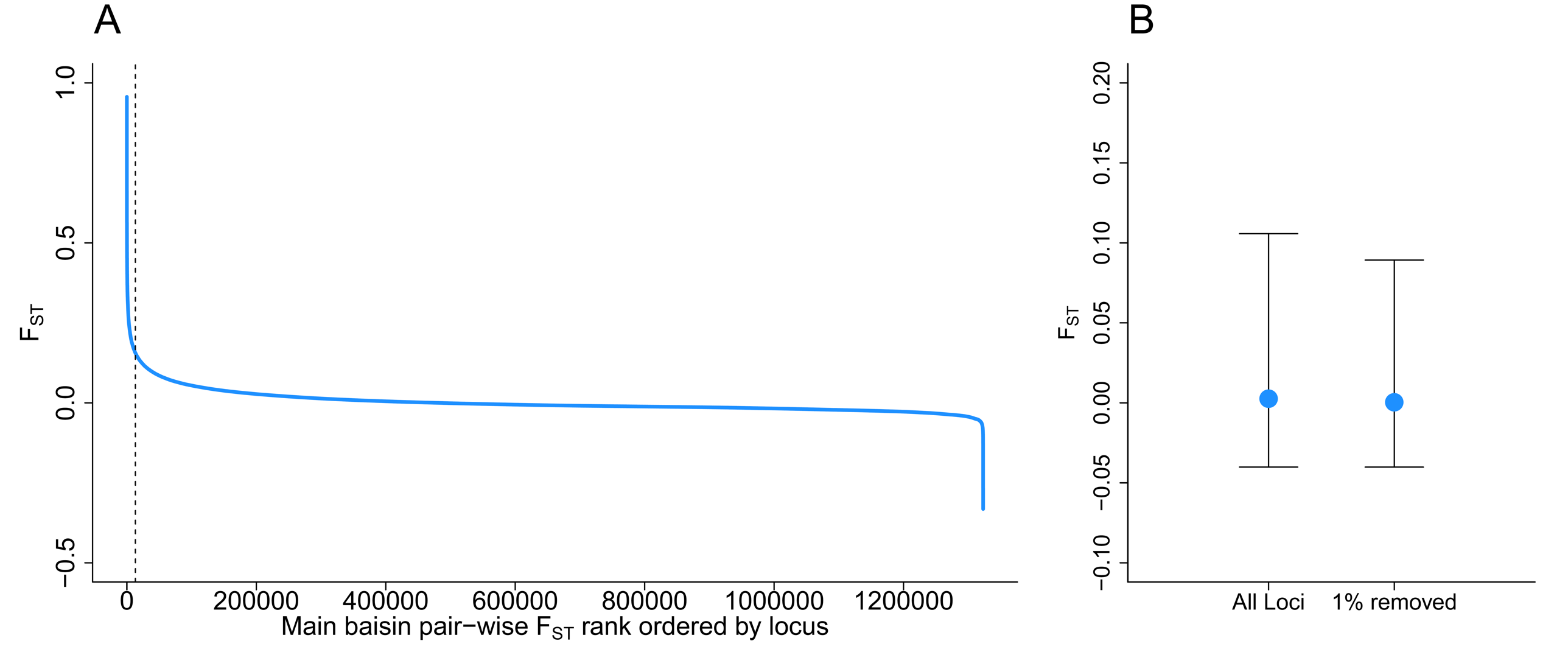


**Figure S21:** Outlier loci did not affect the pair-wise main-basin estimates of *F_ST_*. Panel A illustrates the empirical *F_ST_* values for main-basin comparison per locus (1,323,011 values across all pair-wise comparisons involving the 19 main basin collections; see Table 1). In panel A, all values were rank ordered by *F_ST_* and the top 1% of all values fall to the left of the vertical dashed line. Removing the top 1% of outlier loci across all loci (panel B), resulted in almost no change in empirical estimates of pairwise *F_ST_* values (mean and 95% CI of pairwise *F_ST_* values are plotted). Specifically, removing the top 1% of outlier loci across all loci resulted in a mean change in pairwise *F_ST_* equal to 0.00013 while removing the top 1% within each pair-wise comparison resulted in a mean change in pairwise *F_ST_* of 0.0001. As further confirmation, we compared the northern and southern-most main basin populations (NUB18 and MIC18) using pacadapt (Luu, Bazin, and Blum, 2017). P-values generated from pcadapt were transformed into Bonferroni corrected q-values and a false discovery threshold was set at 10% (alpha = 0.1). Of 9,302 loci, outlier, only 5 loci (0.00054) were identified to deviate from neutral expectation.

**Table S1:** Sampling details including collection site names, site ID, year collected ("Year"), Stage of individuals collected ("yoy = young of year"), Number of individuals collected ("Nc"), Number of individuals genotyped ("Ng"), Sex ratio (male:female), total length (range + SD), and weight (range + SD) for individuals measured. Weight, length, and sex data were not available for some individuals (“na”).

| **Site** | **Site ID** | **Year** | **Stage** | **Nc** | **Ng** | **Sex Ratio** | **Total Length** | **Weight** |
| --- | --- | --- | --- | --- | --- | --- | --- | --- |
| Michigan City | MICYO | 2018 | yoy | 28 | 28 | na | 63 - 104 (7.9) | na |
| Michigan City | MIC19 | 2019 | adult | 50 | 46 | 21:29 | 158-365 (52.5) | 30-740 (180.8) |
| Michigan City | MIC18 | 2018 | adult | 100 | 50 | 22:28 | 128-332 (50.3) | 20-465 (111.3) |
| Saint Joseph | STJ18 | 2018 | adult | 130 | 50 | 45:05:00 | 121-296 (26.9) | 75-255 (45.5) |
| South Haven | SOH18 | 2018 | adult | 18 | 18 | na | 132-256 (33.5) | 20-170 (44.7) |
| South Chicago | SCH18 | 2018 | adult | 16 | 16 | na | 188-247 (18.7) | 62-173 (32.4) |
| Chicago | CHI18 | 2018 | adult | 13 | 13 | na | 145-312 (44.0) | na |
| North Chicago | NCH18 | 2018 | adult | 15 | 15 | na | 147-217(20.5) | na |
| Waukegan | WAK18 | 2018 | adult | 20 | 20 | na | 131-222 (27.4) | na |
| Grand Haven | GRH19 | 2019 | adult | 96 | 38 | na | na | na |
| Grand Haven | GRH18 | 2018 | adult | 109 | 50 | na | 143-225 (19.3) | 30-130 (24.6) |
| Ludington | LUD18 | 2018 | adult | 10 | 10 | 4:06 | 170-228 (16.8) | 20-95 (21.6) |
| Milwaukee | MIL19 | 2019 | adult | 32 | 32 | 15:17 | 166-357 (45.9) | na |
| Algoma | ALG18 | 2018 | adult | 1 | 1 | na | 194 (na) | 125 (na) |
| Suttons Bay | SUT18 | 2018 | adult | 32 | 32 | 4:25 | 190-319 (32.0) | 50-300 (70.36) |
| Northport | NPT18 | 2018 | adult | 34 | 34 | na | na | na |
| Charlevoix | CHX19 | 2019 | adult | 108 | 59 | na | na | na |
| Cheboygan | CHE18 | 2018 | adult | 41 | 40 | 7:34 | 212-269 (16.1) | 100-225 (37.4) |
| Naubinway | NUB18 | 2018 | adult | 72 | 72 | na | na | na |
| Manistique | MAN18 | 2018 | adult | 15 | 15 | na | na | na |
| Grand Haven | BDNYO | 2019 | yoy | 41 | 41 | na | 64-89 (5.7) | na |
| Grand Haven | BDN19 | 2019 | adult | 40 | 40 | na | 102-252 (35.5) | na |
| Little Bay de Noc | LBDYO | 2019 | yoy | 41 | 41 | na | 61 - 74 (3.4) | na |
| Little Bay de Noc | LBD19 | 2019 | adult | 40 | 40 | na | 104-315 (61.6) | na |
| Menominee | MEN19 | 2019 | adult | 49 | 49 | na | 102-264 (41.3) | na |
| South Green Bay | SGB19 | 2019 | adult | 84 | 50 | 34:16:00 | 134-311 (37.6) | na |
| South Green Bay | SGB18 | 2018 | adult | 82 | 60 | 28:32:00 | 100-305 (47.9) | na |

**Table S2:** Genetic diversity within sample sites and regions as measured by observed heterozygosity (*H_o_*), expected heterozygosity (*H_e_*), and allelic richness (*A_r_*). See Table 1 and Figure 1 for site locations.

| **Population** | **Region** | ***H_o_*** | ***H_e_*** | ***A_r_*** |
| --- | --- | --- | --- | --- |
| CHE18 | Main Basin | 0.229 | 0.235 | 1.238 |
| CHI18 | Main Basin | 0.216 | 0.218 | 1.229 |
| CHX19 | Main Basin | 0.234 | 0.231 | 1.234 |
| GRH18 | Main Basin | 0.222 | 0.227 | 1.229 |
| GRH19 | Main Basin | 0.293 | 0.236 | 1.239 |
| LUD18 | Main Basin | 0.222 | 0.212 | 1.228 |
| MAN18 | Main Basin | 0.224 | 0.226 | 1.235 |
| MIC18 | Main Basin | 0.225 | 0.226 | 1.229 |
| MIC19 | Main Basin | 0.226 | 0.226 | 1.228 |
| MICYO | Main Basin | 0.224 | 0.225 | 1.230 |
| MIL19 | Main Basin | 0.223 | 0.225 | 1.229 |
| NCH18 | Main Basin | 0.216 | 0.219 | 1.228 |
| NPT18 | Main Basin | NAN | 0.239 | 1.243 |
| NUB18 | Main Basin | 0.225 | 0.227 | 1.230 |
| SCH18 | Main Basin | 0.214 | 0.216 | 1.225 |
| SOH18 | Main Basin | 0.229 | 0.222 | 1.229 |
| STJ18 | Main Basin | 0.227 | 0.223 | 1.226 |
| SUT18 | Main Basin | 0.232 | 0.230 | 1.234 |
| WAK18 | Main Basin | 0.220 | 0.224 | 1.231 |
| BDN19 | Green Bay | 0.276 | 0.278 | 1.282 |
| BDNYO | Green Bay | 0.279 | 0.279 | 1.283 |
| LBD19 | Green Bay | 0.318 | 0.320 | 1.325 |
| LBDYO | Green Bay | 0.334 | 0.326 | 1.331 |
| MEN19 | Green Bay | 0.279 | 0.284 | 1.287 |
| SGB18 | Green Bay | 0.253 | 0.260 | 1.262 |
| SGB19 | Green Bay | 0.315 | 0.290 | 1.294 |

**Table S3:** Pair-wise estimates of *F_ST_* (Weir and Cockerham's unbiased estimator) for all 9,302 loci and all site comparisons. Numbers in parentheses represent the 95% confidence intervals) Full sample site information (including full site names) can be found in Tables 1 and S1 (Table S3 continued on next page). See Figure 1 for site locations.

|  | BDN19 | BDNYO | CHI18 | WAK18 |
| --- | --- | --- | --- | --- |
| BDN19 | 0 |  |  |  |
| BDNYO | 0.001, (-0.001,0.001) | 0 |  |  |
| CHI18 | 0.08, (0.077,0.082) | 0.081, (0.078,0.083) | 0 |  |
| WAK18 | 0.086, (0.083,0.09) | 0.087, (0.084,0.09) | 0.006, (0.004,0.007) | 0 |
| NCH18 | 0.076, (0.073,0.079) | 0.077, (0.074,0.08) | 0.004, (0.002,0.005) | 0.001, (-0.001,0.003) |
| SCH18 | 0.083, (0.08,0.087) | 0.085, (0.083,0.088) | 0.005, (0.003,0.007) | 0.001, (-0.002,0.002) |
| SGB18 | 0.013, (0.012,0.014) | 0.015, (0.014,0.015) | 0.101, (0.097,0.104) | 0.108, (0.105,0.112) |
| SGB19 | 0.015, (0.014,0.016) | 0.015, (0.014,0.016) | 0.106, (0.102,0.109) | 0.113, (0.11,0.116) |
| GRH18 | 0.093, (0.09,0.096) | 0.094, (0.09,0.097) | 0.006, (0.004,0.007) | 0.001, (-0.001,0.002) |
| GRH19 | 0.088, (0.085,0.091) | 0.089, (0.086,0.092) | 0.013, (0.012,0.015) | 0.007, (0.006,0.008) |
| MIC18 | 0.091, (0.088,0.094) | 0.092, (0.088,0.095) | 0.007, (0.005,0.008) | 0.002, (0.001,0.003) |
| MIC19 | 0.091, (0.087,0.094) | 0.092, (0.089,0.095) | 0.006, (0.004,0.007) | 0.001, (0.001,0.002) |
| LBD19 | 0.022, (0.021,0.023) | 0.023, (0.021,0.024) | 0.115, (0.112,0.118) | 0.123, (0.12,0.126) |
| LBDYO | 0.034, (0.032,0.035) | 0.031, (0.029,0.032) | 0.126, (0.122,0.129) | 0.134, (0.13,0.137) |
| MAN18 | 0.067, (0.064,0.069) | 0.068, (0.065,0.07) | 0.009, (0.007,0.011) | 0.006, (0.005,0.008) |
| CHE18 | 0.077, (0.075,0.08) | 0.078, (0.075,0.081) | 0.007, (0.006,0.009) | 0.004, (0.003,0.005) |
| CHX19 | 0.077, (0.074,0.079) | 0.077, (0.075,0.08) | 0.009, (0.008,0.011) | 0.005, (0.004,0.006) |
| MICYO | 0.088, (0.084,0.091) | 0.089, (0.086,0.092) | 0.005, (0.004,0.007) | 0.001, (0.001,0.002) |
| MEN19 | 0.012, (0.011,0.013) | 0.012, (0.011,0.013) | 0.1, (0.097,0.104) | 0.108, (0.104,0.112) |
| MIL19 | 0.086, (0.083,0.09) | 0.087, (0.084,0.09) | 0.006, (0.004,0.007) | 0.001, (-0.001,0.002) |
| STJ18 | 0.092, (0.088,0.095) | 0.092, (0.089,0.095) | 0.007, (0.006,0.008) | 0.002, (0.002,0.003) |
| LUD18 | 0.083, (0.079,0.086) | 0.084, (0.08,0.088) | 0.007, (0.005,0.009) | 0.002, (-0.001,0.004) |
| SOH18 | 0.086, (0.083,0.089) | 0.088, (0.084,0.091) | 0.007, (0.005,0.008) | -0.001, (-0.002,0.002) |
| SUT18 | 0.073, (0.071,0.076) | 0.075, (0.072,0.078) | 0.01, (0.008,0.011) | 0.006, (0.005,0.007) |
| NPT18 | 0.073, (0.071,0.075) | 0.074, (0.071,0.076) | 0.011, (0.01,0.013) | 0.007, (0.006,0.008) |
| NUB18 | 0.081, (0.078,0.084) | 0.083, (0.08,0.086) | 0.007, (0.006,0.009) | 0.002, (0.002,0.003) |

**Table S3:** Pair-wise estimates of *F_ST_* (Weir and Cockerham's unbiased estimator) for all 9,302 loci and all site comparisons. Numbers in parentheses represent the 95% confidence intervals) Full sample site information (including full site names) can be found in Tables 1 and S1 (Table S3 continued on next page).

|  | NCH18 | SCH18 | SGB18 | SGB19 | GRH18 |
| --- | --- | --- | --- | --- | --- |
| SCH18 | 0.001, (-0.001,0.003) | 0 |  |  |  |
| SGB18 | 0.095, (0.092,0.098) | 0.106, (0.103,0.111) | 0 |  |  |
| SGB19 | 0.101, (0.097,0.104) | 0.112, (0.108,0.115) | 0.006, (0.006,0.007) | 0 |  |
| GRH18 | 0.002, (0.001,0.003) | 0.001, (-0.001,0.002) | 0.113, (0.11,0.117) | 0.121, (0.118,0.125) | 0 |
| GRH19 | 0.008, (0.007,0.01) | 0.008, (0.007,0.009) | 0.109, (0.104,0.112) | 0.115, (0.111,0.118) | 0.004, (0.003,0.004) |
| MIC18 | 0.002, (0.001,0.003) | 0.002, (0.001,0.003) | 0.11, (0.107,0.114) | 0.119, (0.115,0.123) | 0.001, (-0.001,0.001) |
| MIC19 | 0.001, (-0.001,0.002) | 0.001, (-0.001,0.002) | 0.112, (0.108,0.115) | 0.119, (0.115,0.123) | -0.001, (-0.001,0.001) |
| LBD19 | 0.113, (0.11,0.115) | 0.119, (0.116,0.122) | 0.038, (0.036,0.039) | 0.018, (0.017,0.019) | 0.136, (0.133,0.14) |
| LBDYO | 0.124, (0.12,0.127) | 0.13, (0.126,0.134) | 0.048, (0.046,0.05) | 0.024, (0.023,0.025) | 0.148, (0.144,0.151) |
| MAN18 | 0.003, (0.001,0.004) | 0.005, (0.004,0.007) | 0.089, (0.085,0.092) | 0.094, (0.091,0.097) | 0.005, (0.004,0.006) |
| CHE18 | 0.002, (0.001,0.003) | 0.003, (0.002,0.004) | 0.098, (0.095,0.101) | 0.106, (0.102,0.108) | 0.003, (0.002,0.003) |
| CHX19 | 0.004, (0.003,0.005) | 0.004, (0.003,0.005) | 0.096, (0.093,0.099) | 0.106, (0.102,0.109) | 0.004, (0.004,0.004) |
| MICYO | 0.002, (0.001,0.003) | 0.001, (-0.001,0.002) | 0.11, (0.106,0.114) | 0.117, (0.113,0.12) | 0.001, (-0.001,0.001) |
| MEN19 | 0.096, (0.093,0.099) | 0.105, (0.102,0.109) | 0.006, (0.005,0.006) | 0.003, (0.002,0.003) | 0.117, (0.113,0.12) |
| MIL19 | 0.002, (0.001,0.003) | 0.001, (-0.002,0.002) | 0.108, (0.104,0.112) | 0.113, (0.11,0.117) | 0.001, (-0.001,0.001) |
| STJ18 | 0.002, (0.001,0.003) | 0.002, (0.001,0.003) | 0.112, (0.108,0.115) | 0.12, (0.117,0.123) | 0.001, (0.001,0.001) |
| LUD18 | 0.003, (0.001,0.005) | 0.003, (-0.001,0.005) | 0.107, (0.103,0.111) | 0.111, (0.108,0.115) | 0.003, (0.002,0.004) |
| SOH18 | 0.002, (0.001,0.003) | 0.001, (-0.001,0.003) | 0.108, (0.104,0.112) | 0.113, (0.11,0.117) | -0.001, (-0.002,-0.001) |
| SUT18 | 0.003, (0.002,0.004) | 0.005, (0.004,0.006) | 0.096, (0.092,0.099) | 0.103, (0.1,0.106) | 0.005, (0.004,0.005) |
| NPT18 | 0.006, (0.005,0.007) | 0.008, (0.006,0.009) | 0.094, (0.091,0.098) | 0.101, (0.098,0.104) | 0.006, (0.005,0.006) |
| NUB18 | 0.002, (0.001,0.003) | 0.002, (0.001,0.003) | 0.102, (0.099,0.106) | 0.111, (0.108,0.115) | 0.002, (0.001,0.002) |

**Table S3:** Pair-wise estimates of *F_ST_* (Weir and Cockerham's unbiased estimator) for all 9,302 loci and all site comparisons. Numbers in parentheses represent the 95% confidence intervals) Full sample site information (including full site names) can be found in Tables 1 and S1 (Table S3 continued on next page).

|  | GRH19 | MIC18 | MIC19 | LBD19 | LBDYO |
| --- | --- | --- | --- | --- | --- |
| GRH19 | 0 |  |  |  |  |
| MIC18 | 0.004, (0.004,0.005) | 0 |  |  |  |
| MIC19 | 0.004, (0.003,0.005) | 0.001, (-0.001,0.001) | 0 |  |  |
| LBD19 | 0.128, (0.125,0.131) | 0.134, (0.131,0.138) | 0.133, (0.13,0.137) | 0 |  |
| LBDYO | 0.14, (0.136,0.143) | 0.146, (0.142,0.15) | 0.145, (0.142,0.15) | 0.002, (0.002,0.003) | 0 |
| MAN18 | 0.011, (0.01,0.012) | 0.005, (0.004,0.006) | 0.006, (0.005,0.007) | 0.104, (0.101,0.106) | 0.115, (0.112,0.119) |
| CHE18 | 0.005, (0.005,0.006) | 0.003, (0.003,0.004) | 0.003, (0.003,0.004) | 0.119, (0.116,0.122) | 0.131, (0.128,0.134) |
| CHX19 | 0.006, (0.005,0.007) | 0.004, (0.004,0.005) | 0.004, (0.003,0.004) | 0.122, (0.118,0.125) | 0.134, (0.131,0.138) |
| MICYO | 0.005, (0.005,0.006) | 0.001, (0.001,0.002) | 0.002, (0.001,0.002) | 0.128, (0.125,0.132) | 0.14, (0.136,0.143) |
| MEN19 | 0.111, (0.107,0.114) | 0.114, (0.111,0.118) | 0.114, (0.111,0.118) | 0.021, (0.02,0.022) | 0.028, (0.026,0.028) |
| MIL19 | 0.005, (0.005,0.006) | 0.001, (0.001,0.002) | 0.001, (-0.001,0.002) | 0.126, (0.122,0.129) | 0.137, (0.133,0.141) |
| STJ18 | 0.005, (0.004,0.005) | 0.001, (0.001,0.002) | 0.001, (0.001,0.002) | 0.135, (0.132,0.139) | 0.147, (0.144,0.151) |
| LUD18 | 0.012, (0.01,0.013) | 0.003, (0.001,0.005) | 0.003, (0.002,0.004) | 0.115, (0.111,0.118) | 0.126, (0.122,0.129) |
| SOH18 | 0.006, (0.005,0.007) | 0.001, (-0.001,0.002) | 0.001, (-0.001,0.002) | 0.123, (0.12,0.126) | 0.134, (0.131,0.137) |
| SUT18 | 0.007, (0.006,0.008) | 0.005, (0.004,0.005) | 0.005, (0.004,0.006) | 0.116, (0.112,0.119) | 0.128, (0.124,0.131) |
| NPT18 | 0.008, (0.007,0.008) | 0.006, (0.005,0.007) | 0.006, (0.005,0.007) | 0.115, (0.112,0.118) | 0.127, (0.123,0.13) |
| NUB18 | 0.005, (0.004,0.005) | 0.002, (0.002,0.002) | 0.002, (0.001,0.002) | 0.127, (0.123,0.13) | 0.139, (0.135,0.143) |

**Table S3:** Pair-wise estimates of *F_ST_* (Weir and Cockerham's unbiased estimator) for all 9,302 loci and all site comparisons. Numbers in parentheses represent the 95% confidence intervals) Full sample site information (including full site names) can be found in Tables 1 and S1 (Table S3 continued on next page).

|  | MAN18 | CHE18 | CHX19 | MICYO | MEN19 |
| --- | --- | --- | --- | --- | --- |
| MAN18 | 0 |  |  |  |  |
| CHE18 | -0.001, (-0.002,0.001) | 0 |  |  |  |
| CHX19 | 0.001, (-0.001,0.002) | 0.001, (0.001,0.002) | 0 |  |  |
| MICYO | 0.005, (0.004,0.006) | 0.003, (0.002,0.004) | 0.005, (0.004,0.006) | 0 |  |
| MEN19 | 0.089, (0.085,0.092) | 0.101, (0.098,0.104) | 0.101, (0.098,0.104) | 0.112, (0.108,0.115) | 0 |
| MIL19 | 0.005, (0.004,0.006) | 0.003, (0.002,0.004) | 0.004, (0.003,0.005) | 0.001, (-0.001,0.001) | 0.109, (0.105,0.113) |
| STJ18 | 0.007, (0.006,0.008) | 0.004, (0.003,0.004) | 0.004, (0.004,0.005) | 0.001, (0.001,0.002) | 0.115, (0.111,0.118) |
| LUD18 | 0.008, (0.006,0.01) | 0.005, (0.003,0.006) | 0.006, (0.005,0.007) | 0.004, (0.002,0.005) | 0.105, (0.101,0.108) |
| SOH18 | 0.005, (0.004,0.007) | 0.002, (0.001,0.003) | 0.004, (0.004,0.005) | -0.001, (-0.001,0.001) | 0.108, (0.105,0.111) |
| SUT18 | 0.001, (-0.001,0.002) | 0.001, (-0.001,0.002) | -0.001, (-0.001,0.001) | 0.005, (0.004,0.006) | 0.098, (0.095,0.101) |
| NPT18 | 0.003, (0.002,0.004) | 0.002, (0.002,0.003) | 0.002, (0.002,0.002) | 0.006, (0.005,0.007) | 0.097, (0.094,0.1) |
| NUB18 | 0.002, (0.001,0.003) | 0.001, (-0.001,0.001) | 0.001, (0.001,0.002) | 0.002, (0.002,0.003) | 0.106, (0.103,0.11) |

**Table S3:** Pair-wise estimates of *F_ST_* (Weir and Cockerham's unbiased estimator) for all 9,302 loci and all site comparisons. Numbers in parentheses represent the 95% confidence intervals) Full sample site information (including full site names) can be found in Tables 1 and S1.

|  | MIL19 | STJ18 | LUD18 | SOH18 | SUT18 | NPT18 | NUB18 |
| --- | --- | --- | --- | --- | --- | --- | --- |
| MIL19 | 0 |  |  |  |  |  |  |
| STJ18 | 0.002, (0.001,0.002) | 0 |  |  |  |  |  |
| LUD18 | 0.005, (0.003,0.006) | 0.005, (0.004,0.007) | 0 |  |  |  |  |
| SOH18 | -0.001, (-0.001,0.001) | 0.001, (0.001,0.002) | 0.003, (0.001,0.005) | 0 |  |  |  |
| SUT18 | 0.004, (0.004,0.005) | 0.006, (0.005,0.006) | 0.007, (0.005,0.009) | 0.005, (0.004,0.006) | 0 |  |  |
| NPT18 | 0.006, (0.006,0.007) | 0.007, (0.006,0.007) | 0.012, (0.01,0.014) | 0.007, (0.007,0.008) | 0.001, (-0.001,0.002) | 0 |  |
| NUB18 | 0.002, (0.001,0.003) | 0.002, (0.002,0.003) | 0.005, (0.003,0.007) | 0.001, (0.001,0.002) | 0.001, (0.001,0.002) | 0.003, (0.002,0.003) | 0 |

**Table S4:** Results of analyses of all yellow perch RadSeq data with NewHybrids. Each row represents a sample site (Site ID), the sample size (N indiv), the number of individuals that were identified as a hybrid (N hyb) based on a cutoff of individual assignments with a greater or equal to 0.5 posterior probability, and the percentage of “hybrids” within each population (% hyb). Full location details for Site ID can be found in Figure 1 and Table 1.

| **Site ID** | **Region** | **N indiv** | **N hyb** | **% hyb** |
| --- | --- | --- | --- | --- |
| BDN19 | Green Bay | 37 | 5 | 13.5 |
| BDNYO | Green Bay | 41 | 5 | 12.2 |
| LBD19 | Green Bay | 39 | 2 | 5.1 |
| LBDYO | Green Bay | 40 | 0 | 0.0 |
| MEN19 | Green Bay | 49 | 4 | 8.2 |
| SGB18 | Green Bay | 60 | 5 | 8.3 |
| SGB19 | Green Bay | 48 | 4 | 8.3 |
|  | **total=** | **314** | **25** | **8.0** |
| CHI18 | Main Basin | 12 | 1 | 8.3 |
| WAK18 | Main Basin | 20 | 0 | 0.0 |
| NCH18 | Main Basin | 15 | 0 | 0.0 |
| SCH18 | Main Basin | 14 | 0 | 0.0 |
| GRH18 | Main Basin | 50 | 0 | 0.0 |
| GRH19 | Main Basin | 38 | 1 | 2.6 |
| MIC18 | Main Basin | 49 | 0 | 0.0 |
| MIC19 | Main Basin | 46 | 0 | 0.0 |
| MAN18 | Main Basin | 14 | 1 | 7.1 |
| CHE18 | Main Basin | 40 | 0 | 0.0 |
| CHX19 | Main Basin | 58 | 0 | 0.0 |
| MICYO | Main Basin | 28 | 1 | 3.6 |
| MIL19 | Main Basin | 32 | 2 | 6.3 |
| STJ18 | Main Basin | 49 | 0 | 0.0 |
| LUD18 | Main Basin | 8 | 0 | 0.0 |
| SOH18 | Main Basin | 17 | 0 | 0.0 |
| SUT18 | Main Basin | 32 | 0 | 0.0 |
| NPT18 | Main Basin | 34 | 0 | 0.0 |
| NUB18 | Main Basin | 57 | 0 | 0.0 |
|  | **total=** | **613** | **6** | **1.0** |
